# Psychological pathways linking body mass index and depression in women and men in a Colombian sociodemographic context

**DOI:** 10.3389/fpsyg.2026.1980836

**Published:** 2026-10-29

**Authors:** Duban Romero, Valentina Salazar, Cipriano Quiros, Guillermo Villarino Martínez, Viviana Ahumada, Jesús Amaranto, Moisés Mebarak

**Affiliations:** 1 Faculty of Statistical Studies, Universidad Complutense de Madrid, Madrid, Spain; 2 Psychology Department, Universidad Sergio Arboleda, Bogotá, Colombia; 3 Faculty of Education, Universidad Internacional de La Rioja, Logroño, Spain; 4 Psychology Department, Universidad del Norte, Barranquilla, Colombia

**Keywords:** body mass index, body self-esteem, depressive symptoms, eating disorder symptoms, gender differences, mediation analysis, structural equation modeling, young adults

## Abstract

Body mass index (BMI) shows inconsistent associations with depressive symptoms, and it remains unclear whether body self-esteem and eating disorder symptoms mediate this association, and whether this mediation differs by gender. Therefore, this study used multigroup structural equation modeling to examine these mediators in Colombian young adults, separately for women and men. A total of 662 participants aged 18 to 30 years (398 women, 60.1%; Mage = 21.75, SD = 3.11) were recruited through snowball sampling and completed an online survey with self-reported weight and height and validated measures of body self-esteem, eating disorder symptoms and depressive symptoms. BMI showed no total association with depressive symptoms in women or men. Nevertheless, higher BMI was associated with lower body self-esteem and more eating disorder symptoms, and both mediators were associated with more depressive symptoms, yielding positive indirect effects in women (*b* = 0.033, 95% CI [0.022, 0.045]) and men (*b* = 0.015, 95% CI [0.006, 0.028]). In men, mediation was indirect-only, whereas in women a negative direct effect offset the indirect effect, a pattern of competitive mediation. The path from BMI to eating disorder symptoms was stronger in women, but the relative weight of the two pathways did not differ by gender. Body self-esteem and eating disorder symptoms link BMI to depressive symptoms in both women and men, with quantitative rather than qualitative gender differences, which suggests that both processes deserve attention in prevention and intervention efforts with young adults.

## Introduction

Mental health problems are a leading public health challenge among adolescents and young adults. The World Health Organization (2025) estimates that approximately one in seven adolescents lives with a mental disorder, and a meta-analysis of 72 studies concluded that about one third of adolescents worldwide report elevated depressive symptoms, a proportion that rose from 24% in 2001 to 2010 to 37% in 2011 to 2020 (Shorey et al., 2022). Anxiety follows a similar pattern: during the first year of the COVID-19 pandemic, the pooled prevalence of clinically elevated depressive and anxiety symptoms among children and adolescents was 25.2 and 20.5%, respectively, roughly double the pre-pandemic estimates (Racine et al., 2021). Depression, anxiety and eating disorders also tend to co-occur, since anxiety and mood disorders are among the most frequent psychiatric comorbidities of eating disorders (Hambleton et al., 2022). Although anxiety is therefore an important part of this landscape, the present study concentrates on depression and eating disorder symptoms because both are closely tied to the way people evaluate their own bodies, the psychological process through which body weight may translate into emotional distress.

Eating disorders have also become a growing concern. Population data indicate that the risk of bulimia nervosa and binge eating disorder has increased across successive birth cohorts (Hudson et al., 2007), and a systematic review of 53 studies reported a large increase in hospitalizations and in eating disorder symptoms after the onset of the COVID-19 pandemic, together with higher anxiety and depression among patients (Devoe et al., 2023). The explanations proposed for this increase include the loss of daily structure and routines, greater social isolation, the negative influence of media and reduced access to care during lockdowns (Devoe et al., 2023). In Latin America, the epidemiology of eating disorders has been the subject of a recent systematic review (Kolar and Mebarak, 2022), but evidence that relates these problems to body weight and depression within a single model remains scarce.

The relations among body image, eating behavior and depression appear to be multidimensional rather than linear and unidirectional. In adolescents with type 1 diabetes, for instance, the direct effect of BMI on eating disorders was not significant, whereas indirect effects through body dissatisfaction, alone and followed by depression or by self-esteem and depression, were significant (Park and Ju, 2021). Among people aged 15–25 years, perceived body image dissatisfaction was associated with lower self-esteem and with higher depression, anxiety and abnormal eating attitudes (Malik and Ali, 2021). In adults, depression and higher BMI contribute independently to body image dissatisfaction (Paans et al., 2018), which is consistent with the reciprocal associations reported between body dissatisfaction and depressive symptoms from adolescence to early adulthood (Sharpe et al., 2018). Longitudinal studies also identify body dissatisfaction as a risk factor for disordered eating, both in overweight adolescents and young adults (Goldschmidt et al., 2015) and over 10 years from adolescence to young adulthood (Foster et al., 2023). Taken together, these findings suggest that body-related self-evaluation, disordered eating and depressive symptoms overlap clinically and share vulnerability factors, so that they are better studied jointly than in isolation.

Evidence on the association between BMI and depression is not straightforward. A meta-analysis of longitudinal studies concluded that obesity increases the risk of depression (OR = 1.55) and overweight does so to a lesser extent (OR = 1.27), the latter association being significant in adults but not in people younger than 20 years (Luppino et al., 2010). Cross-sectional findings are less consistent: in a national survey of 43,534 Dutch adults aged 18–90 years, the association followed a U-shaped rather than a linear pattern, with elevated depression at both ends of the BMI distribution, and there was a trend toward a difference between men and women (de Wit et al., 2009). These heterogeneous results suggest that psychological variables, rather than weight alone, may determine when BMI relates to depression.

Consistent with this idea, in 397 Chilean adolescents, body dissatisfaction was positively correlated with BMI *z*-score and depression and negatively with self-esteem (Morán et al., 2024). Mediation studies point in the same direction: perceived overweight, body image dissatisfaction and weight-based rejection sensitivity were tested as mediators between BMI and depressive symptoms in 607 Taiwanese adults (Lin et al., 2025), body esteem mediated the link between childhood obesity and depressive symptoms 5 years later (Beltrán-Garrayo et al., 2024), and longitudinal pathways from childhood BMI to adolescent depression through body dissatisfaction have been examined in a large United Kingdom cohort (Blundell et al., 2024). Most of these studies, however, analyzed BMI and depression or BMI and eating disorders separately, in adolescents, in clinical samples or outside Latin America, and did not integrate body self-esteem and eating disorder symptoms as simultaneous mediators.

Gender may also shape these processes. Body dissatisfaction is higher in women than in men and, in women, does not vary with age (Quittkat et al., 2019). Sociocultural ideals differ as well: contemporary standards for women emphasize thinness, whereas those for men emphasize a muscular body shape (Tiggemann, 2004), which may lead women to express body-related distress more often through eating-related concerns and men through broader evaluations of the body. Consistent with this, body dissatisfaction partially explained gender differences in depressive symptoms and disordered eating during adolescence (Ferreiro et al., 2014), and sex differences have been reported in the relationships between body dissatisfaction, quality of life and psychological distress (Griffiths et al., 2016). The evidence is not uniform, however: body esteem mediated the association between childhood obesity and adolescent depressive symptoms equally in boys and girls (Beltrán-Garrayo et al., 2024). Whether the mechanisms linking BMI and depression differ between women and men therefore remains an open question, particularly in Latin American young adults.

The present study examined a multigroup mediation model in Colombian young adults aged 18–30 years, in which BMI, from normal weight to obesity, is related to depressive symptoms through body self-esteem and eating disorder symptoms simultaneously, separately for women and men. Based on the evidence reviewed, we tested two hypotheses:

*H1*. Higher BMI will be associated with lower body self-esteem and with more eating disorder symptoms, and both mediators will be associated with more depressive symptoms, so that BMI will show indirect effects on depressive symptoms through each mediator in women and in men.

*H2*. The indirect effect through eating disorder symptoms will be stronger in women than in men, whereas the indirect effect through body self-esteem will be stronger in men than in women.

## Methods

### Sample and procedure

The survey was addressed to Colombian adults (18 years or older) who had already started their sexual life, because it was part of a larger study that also included tests on sexual dynamics. Participants were recruited through snowball sampling, initiated with psychology students from private universities in the Colombian Caribbean region, who forwarded the survey to individuals outside their universities. This strategy is widely used in behavioral and health research when probability sampling is not feasible (Sadler et al., 2010). Data were collected through an online Qualtrics survey. At the beginning of the survey, participants read and accepted an informed consent form, and responses that failed an attention-check item were excluded before the analysis. The study was approved by the ethics committee of Universidad del Norte [N° 332].

The processed data file included 766 participants. Analyses were restricted to participants aged 18–30 years because eating disorders typically emerge in late adolescence and early adulthood (Hudson et al., 2007), and because appearance-related concerns and eating disorder symptomatology tend to decline with age (Quittkat et al., 2019; Tiggemann and Lynch, 2001), so that the processes examined here may differ in older adults. This criterion excluded 48 participants older than 30 years and five who did not report their age. Participants classified as underweight (BMI ≤ 18.5 kg/m^2^; *n* = 45, 6.3% of the age-eligible participants) were excluded from all analyses. Because the association between BMI and depression has been described as U-shaped, with elevated depression at both ends of the BMI distribution (de Wit et al., 2009), including these participants could have introduced a nonlinear component that a linear specification cannot capture. Therefore, the study was restricted to a BMI from 18.5 kg/m^2^ upward, from the normal weight to the obesity range. Finally, six women with a BMI above 50 kg/m^2^ (range 52.9–66.4) were excluded from the analytic sample. This decision was taken after influence diagnostics showed that these valid observations exerted a disproportionate influence on the estimated relations between BMI and the mediators. Results, including them, are reported in full in the Supplementary Materials.

The analytic sample comprised 662 participants, of whom 398 were women (60.1%) and 264 were men (39.9%) according to the gender they reported, with a mean age of 21.75 years (SD = 3.11, range 18–30). Regarding educational levels, 55.9% had enrolled in university studies. 47.9% of participants reported being in a committed relationship and 52.1% being single. According to BMI (*M* = 24.37, SD = 4.08, range 18.5–49.6 kg/m^2^), 64.0% were within the normal range, 27.3% were overweight and 8.6% had obesity. Missing data were handled with full information maximum likelihood (FIML): 23 participants (3.5%) did not answer any PHQ-9 item, and there were no missing data in the remaining variables. A sensitivity analysis indicated that, with *α* = 0.05 and 80% power, the sample could detect correlations of at least 0.14 in women and 0.17 in men (see Supplementary Materials).

### Measures

#### Body mass index

Participants self-reported their weight and height in the survey, and BMI was calculated as weight in kilograms divided by height in meters squared. It was analyzed as a continuous predictor (kg/m^2^) and, for descriptive purposes, classified as normal weight (18.5 to 25), overweight (25–30) and obesity (above 30 kg/m^2^).

*Body self-esteem*. The Body Esteem Scale for Adolescents and Adults (BESAA; Mendelson et al., 2001), in its Latin American version (BESAA-LA; Andres et al., 2023), assesses evaluations of one’s body and appearance. It has 18 items rated on a 5-point scale (1 = never to 5 = always) that form three subscales: weight satisfaction, negative appearance and positive appearance. Higher scores on the global body self-esteem score indicate higher body self-esteem. In this sample, omega was 0.90 for weight satisfaction, 0.76 for negative appearance and 0.93 for positive appearance (see Supplementary Materials).

#### Eating disorder symptoms

The Eating Disorder Examination Questionnaire 8 (EDE-Q8; Kliem et al., 2016) is the abbreviated version of the EDE-Q (Fairburn and Beglin, 1994). Its eight items assess eating disorder psychopathology during the past 4 weeks, and respondents indicate the number of days on which each concern or behavior occurred, from “no days” to “every day.” Higher scores indicate more severe symptoms. The scale was culturally adapted and its psychometric properties were evaluated for Colombian participants in this study; a one-factor model with residual covariances between items 4 and 5 and between items 7 and 8 was retained, and omega was 0.86 (see Supplementary Materials).

#### Depressive symptoms

The Patient Health Questionnaire-9 (PHQ-9; Kroenke et al., 2001), in the version validated in Colombia (Cassiani-Miranda et al., 2021), has nine items based on the DSM-5 criteria for depression (American Psychiatric Association, 2013). Items are rated on a 4-point scale (0 = not at all to 3 = nearly every day) according to the frequency of symptoms over the past 2 weeks, and higher scores indicate more depressive symptoms. Omega was 0.87 in this sample.

#### Data analysis

Analyses were conducted in R (version 4.5.2; R Core Team, 2025) with the *lavaan* (Rosseel et al., 2025).

##### Measurement models and scores

The psychometric properties of the scales were evaluated with confirmatory factor analysis (CFA) estimated with robust maximum likelihood (MLR) and FIML. Fit was assessed with the robust CFI, TLI and RMSEA and the SRMR, using conventional cutoffs (CFI and TLI > 0.90; RMSEA <0.08), and reliability was estimated with McDonald’s omega. All scores used in the analyses were factor scores derived from the confirmatory models: the sample was randomly divided into two halves, the first used for exploratory factor analysis and the second for CFA, and the factor scores of all participants were then predicted from the CFA solutions and standardized (*M* = 50, SD = 10). Measurement invariance of the PHQ-9 between women and men was tested with multigroup CFA (configural, metric and scalar models), judging changes in fit with the criteria of Chen (2007).

##### Descriptive and bivariate analyses

Medians and interquartile ranges were computed by BMI category, and differences were tested with the Kruskal-Wallis test because the scores are not strictly parametric. The effect size was the rank epsilon-squared, *H*/(*n* − 1) (Tomczak and Tomczak, 2014), and pairwise comparisons used Wilcoxon rank-sum tests with Holm correction. Associations were estimated with Spearman correlations, with confidence intervals based on Fisher’s transformation (Bonett and Wright, 2000) and *p*-values adjusted with the Holm method.

##### Mediation model

A multigroup structural equation model was specified in which BMI predicted body self-esteem and eating disorder symptoms (paths a1 and a2) and depressive symptoms (direct path c′), and both mediators predicted depressive symptoms (paths b1 and b2) and were allowed to covary. Depressive symptoms were modeled as a latent variable with the nine PHQ-9 items as indicators, whereas the mediators entered as standardized factor scores. The model was estimated separately for women and men with MLR and FIML. Specific indirect effects were computed as the products a1 × b1 and a2 × b2, the total indirect effect as their sum, and the total effect as the sum of the direct and total indirect effects. Because the distribution of a product of coefficients is not normal, 95% confidence intervals for the indirect and total effects were obtained with the percentile bootstrap (5,000 resamples; Preacher and Hayes, 2008). Following Zhao et al. (2010), the pattern of mediation was classified as complementary, competitive or indirect-only according to the significance and sign of the direct and indirect effects, without requiring a significant total effect. Unstandardized coefficients (*b*) are expressed in the metric of the variables (BMI in kg/m^2^, mediators in standardized points), and fully standardized coefficients (*β*) are also reported.

##### Comparison between women and men

Differences between groups were examined with Wald tests of the differences in each path and in the indirect and total effects (delta method with robust standard errors), including a contrast between the two specific indirect effects within each group and the difference of these contrasts between groups. In addition, scaled chi-square difference tests compared the freely estimated model with models in which paths were constrained to be equal across groups. The omnibus test of equality of all structural paths was considered the primary test, and the tests of individual contrasts were considered exploratory.

##### Sensitivity analyses

To examine robustness, the following analyses are reported in Supplementary Materials: (a) a model with a residual covariance between PHQ-9 items 3 and 4; (b) a model with all constructs specified as latent variables; (c) the structural model under metric and scalar measurement constraints; and (d) analyses of extreme BMI values, including influence diagnostics (leverage, Cook’s distance and DFBETAS; Belsley et al., 1980), tests of linearity, the model under alternative BMI cutoffs and with a log-transformed BMI. Statistical power was addressed with a sensitivity analysis of the smallest detectable effects instead of *post hoc* power (Hoenig and Heisey, 2001).

## Results

### Descriptive statistics and bivariate associations

Table 1 shows the scores by BMI category. Body self-esteem (*H* = 46.60, df = 2, *p* < 0.001, *ε^2^* = 0.071) and eating disorder symptoms (*H* = 39.23, *p* < 0.001, *ε^2^* = 0.059) differed across BMI categories, whereas depressive symptoms did not (*H* = 0.77, *p* = 0.682, *ε^2^* = 0.001). Higher BMI categories showed lower body self-esteem, which differed between all pairs of categories (all Holm-adjusted *p* < 0.001), and more eating disorder symptoms, which were higher in the overweight and obesity categories than in the normal weight category (both *p* < 0.001) but did not differ between overweight and obesity (*p* = 0.147; see Table 1).

**Table 1:** Descriptive statistics by BMI.

Variable	General (*N* = 662)	Normal weight (*n* = 424)	Overweight (*n* = 181)	Obesity (*n* = 57)	*H*	*p*	*ε^2^*
Depressive	47.53 (12.43)	47.11 (11.99)	48.01 (12.86)	48.46 (13.02)	0.77	0.682	0.001
Eating disorders	46.70 (13.87)	44.86 (10.17)	51.57 (19.32)	54.66 (18.32)	39.23	<0.001	0.059
Body self-esteem	50.54 (13.35)	52.10 (11.97)	48.86 (14.35)	42.65 (11.03)	46.60	<0.001	0.071

Scores are standardized (*M* = 50, SD = 10). Kruskal-Wallis tests, df = 2.

Table 2 presents the Spearman correlations for women (below the diagonal) and men (above the diagonal). In both groups, BMI was positively associated with eating disorder symptoms (*ρ* = 0.37 in women and 0.26 in men) and negatively with body self-esteem (*ρ* = −0.21 and −0.17), but it was not associated with depressive symptoms (women: *ρ* = 0.01, 95% CI [−0.09, 0.11]; men: *ρ* = 0.01, 95% CI [−0.12, 0.13]). Depressive symptoms correlated positively with eating disorder symptoms (*ρ* = 0.45 and 0.31) and negatively with body self-esteem (*ρ* = −0.46 and −0.33), and the two mediators were strongly and negatively correlated (*ρ* = −0.59 and −0.49), which was accommodated in the structural model through their covariance.

**Table 2:** Spearman correlations for women (below the diagonal) and men (above the diagonal).

Feature	1	2	3	4
1. BMI	–	0.01	0.26^***^	−0.17^**^
2. Depressive	0.01	–	0.31^***^	−0.33^***^
3. Eating disorders	0.37^***^	0.45^***^	–	−0.49^***^
4. Body self-esteem	−0.21^***^	−0.46^***^	−0.59^***^	–

Women: *n* up to 398; men: *n* up to 264. ^**^p < 0.01, ^***^p < 0.001 (Holm-adjusted).

### Mediation model

The model showed acceptable fit according to CFI, TLI and SRMR and marginal fit according to RMSEA, *χ^2^* (102) = 288.21, CFI = 0.924, TLI = 0.902, RMSEA = 0.083, 90% CI [0.072, 0.095], SRMR = 0.048. Adding a residual covariance between PHQ-9 items 3 and 4 improved fit (RMSEA = 0.074, CFI = 0.941, TLI = 0.922) without changing the estimates. Among women, higher BMI was associated with more eating disorder symptoms (*b* = 1.007, 95% CI [0.706, 1.309], *β* = 0.358, *p* < 0.001) and lower body self-esteem (*b* = −0.672, 95% CI [−0.899, −0.444], *β* = −0.265, *p* < 0.001). In turn, lower body self-esteem (*b* = −0.018, *β* = −0.300) and more eating disorder symptoms (*b* = 0.020, *β* = 0.367) were associated with more depressive symptoms (both *p* < 0.001). After accounting for the mediators, BMI had a negative direct effect on depressive symptoms (*b* = −0.028, 95% CI [−0.044, −0.012], *β* = −0.181, *p* < 0.001). Both specific indirect effects were positive, through body self-esteem (*b* = 0.012, bootstrap 95% CI [0.006, 0.020]) and through eating disorder symptoms (*b* = 0.020, [0.011, 0.032]), and so was the total indirect effect (*b* = 0.033, [0.022, 0.045]). Because the positive indirect effect and the negative direct effect were of similar magnitude, the total effect of BMI on depressive symptoms did not differ from zero (*b* = 0.005, [−0.012, 0.022]), which corresponds to competitive mediation (see Figure 1).

**Figure 1:**
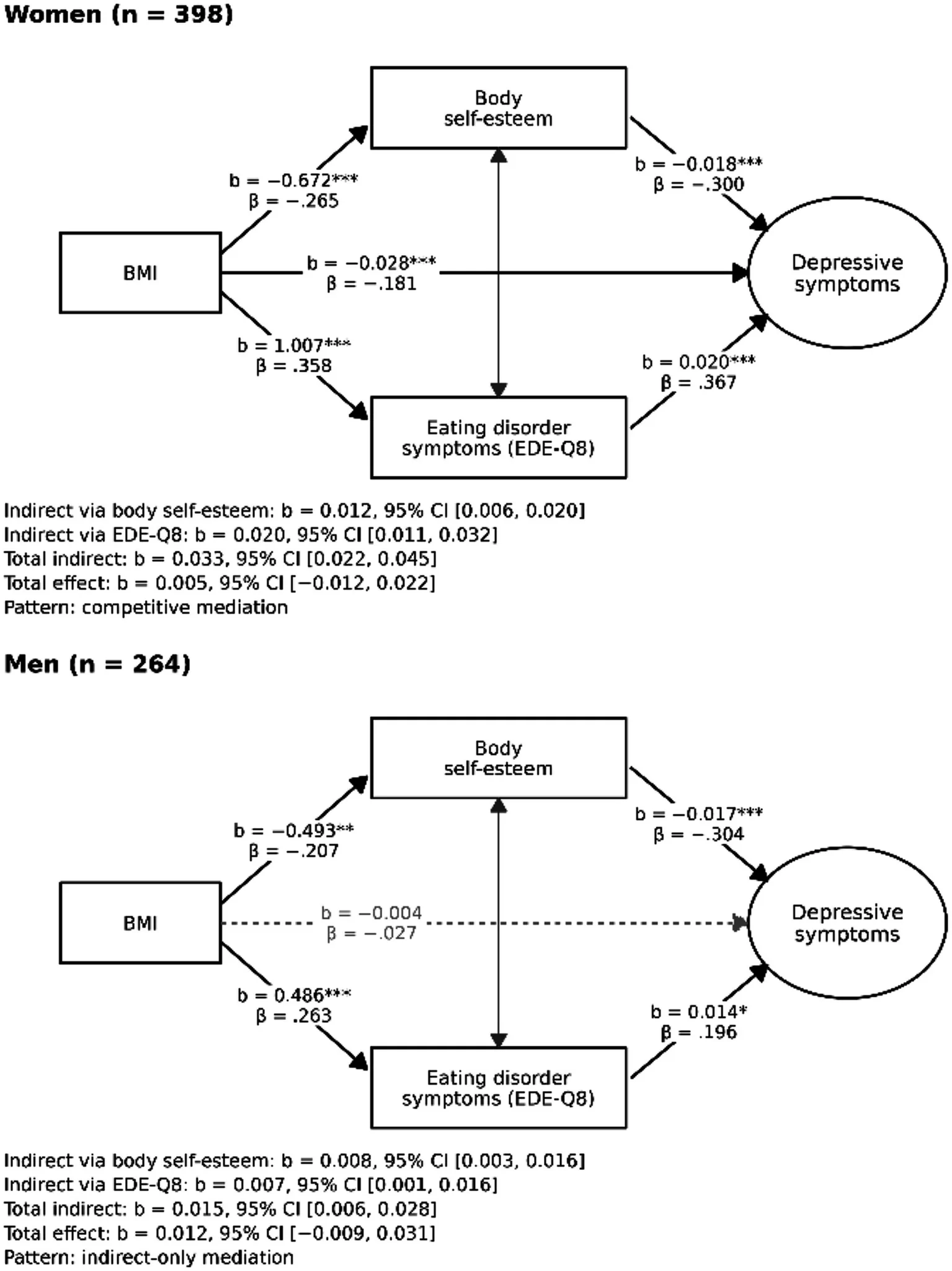
Direct, indirect and total effects of BMI on depressive symptoms. Values on the arrows are unstandardized coefficients (*b*; BMI in kg/m^2^) and, below them, fully standardized coefficients (*β*). ^*^p < 0.05, ^**^p < 0.01, ^***^p < 0.001; dashed gray arrows indicate non-significant paths. The double-headed arrow represents the covariance between the mediators, which was estimated freely. Depressive symptoms were modeled as a latent variable with nine PHQ-9 indicators (not shown). Confidence intervals of the indirect and total effects are percentile bootstrap intervals (5,000 resamples).

Among men, higher BMI was associated with lower body self-esteem (*b* = −0.493, 95% CI [−0.793, −0.192], *β* = −0.207, *p* = 0.001) and more eating disorder symptoms (*b* = 0.486, [0.215, 0.757], *β* = 0.263, *p* < 0.001), and both mediators predicted depressive symptoms (*b* = −0.017, *β* = −0.304, *p* < 0.001, and *b* = 0.014, *β* = 0.196, *p* = 0.010). The direct effect was not significant (*b* = −0.004, [−0.027, 0.020], *p* = 0.765). The specific indirect effects through body self-esteem (*b* = 0.008, bootstrap 95% CI [0.003, 0.016]) and through eating disorder symptoms (*b* = 0.007, [0.001, 0.016]) and the total indirect effect (*b* = 0.015, [0.006, 0.028]) were positive, whereas the total effect was not significant (*b* = 0.012, [−0.009, 0.031]), which corresponds to indirect-only mediation. In both groups the indirect effects were small in standardized terms (total indirect *β* = 0.211 in women and 0.115 in men), and the total effect agreed with that of a model without mediators (women *b* = 0.005, *p* = 0.544; men *b* = 0.011, *p* = 0.283).

### Differences between women and men

Constraining all structural paths to be equal across groups worsened model fit, Δ*χ^2^* (5) = 12.34, *p* = 0.030, ΔCFI = −0.003. The difference was concentrated in the paths from BMI to the mediators (Δ*χ^2^* (2) = 8.44, *p* = 0.015), whereas the paths from the mediators to depressive symptoms (Δ*χ^2^* (2) = 1.29, *p* = 0.525) and the direct path (Δ*χ^2^* (1) = 3.14, *p* = 0.076) did not differ significantly. The path from BMI to eating disorder symptoms was stronger in women than in men (difference in *b* = 0.521, 95% CI [0.116, 0.926], *p* = 0.012), and the indirect effect through eating disorder symptoms (difference = 0.013, [0.001, 0.026], *p* = 0.035) and the total indirect effect (difference = 0.017, [0.002, 0.033], *p* = 0.029) were larger in women. These contrasts are exploratory, and none remained significant after Holm correction across the nine principal contrasts (smallest adjusted *p* = 0.108). The relative weight of the two indirect pathways did not differ between groups: the contrast between the indirect effect through eating disorder symptoms and through body self-esteem was 0.008 in women (*p* = 0.231) and −0.002 in men (*p* = 0.731), and the difference between these contrasts was 0.009 (95% CI [−0.006, 0.025], *p* = 0.234).

### Sensitivity analyses

Six women were excluded because they had BMI values from 52.9 to 66.4 kg/m^2^ (*z* scores from 5.3 to 7.8). All six were flagged as influential in the regressions of the mediators on BMI, and their inclusion flattened the slopes of BMI on eating disorder symptoms (from 1.007 to 0.350) and on body self-esteem (from −0.672 to −0.204) in women (see Supplementary Materials). When they were included, the direct effect in women was no longer significant (*b* = −0.011, *β* = −0.108, *p* = 0.076), the pattern became indirect-only mediation, and equality of all paths across groups was not rejected (Δ*χ^2^* (5) = 2.94, *p* = 0.709). The results in women were stable when the exclusion cutoff varied between 40 and 50 kg/m^2^ (*β* of the direct effect between −0.181 and −0.182; *β* of the total indirect effect between 0.211 and 0.218), and competitive mediation was preserved with cutoffs up to 60 kg/m^2^. Finally, the relation between BMI and eating disorder symptoms was concave in both groups (quadratic term *p* < 0.001 in women and *p* = 0.023 in men), so the linear coefficients should be read as approximations over the observed BMI range (see Supplementary Materials).

## Discussion

This study examined whether body self-esteem and eating disorder symptoms link BMI to depressive symptoms in Colombian young adults, separately for women and men. Three findings stand out. First, BMI was not associated with depressive symptoms at the bivariate level or in terms of its total effect, in either group. Second, BMI was nevertheless associated with lower body self-esteem and with more eating disorder symptoms, and both mediators were associated with more depressive symptoms, which produced positive indirect effects in women and in men. Third, the paths from BMI to the mediators differed between groups, particularly the path to eating disorder symptoms, which was stronger in women, whereas the relative weight of the two indirect pathways did not differ between women and men.

The absence of a total effect indicates that the results should not be described as full mediation. In men, the pattern corresponds to indirect-only mediation: the indirect effect was significant and the direct effect was not, without a significant total effect. In women, the pattern was competitive: the positive indirect effect (*b* = 0.033) was offset by a negative direct effect (*b* = −0.028), so that the net association was close to zero. As Zhao et al. (2010) note, a significant indirect effect does not require a significant total effect, but the competitive pattern in women implies that other processes linked to BMI, and not measured here, operate in the opposite direction to the pathways examined. The direct effect is a residual association net of the mediators and should not be interpreted causally.

This result agrees with the inconsistency of the literature on BMI and depression. Longitudinal evidence indicates that obesity increases the risk of depression (Luppino et al., 2010), whereas cross-sectional evidence has reported nonlinear associations (de Wit et al., 2009), and our data show no bivariate association within the BMI range studied. The additional analyses show no evidence of curvature between BMI and depressive symptoms in women (quadratic term *p* = 0.847) and only a marginal one in men (*p* = 0.081), although this conclusion is limited to a range that starts at 18.5 kg/m^2^. What the data do show is that BMI is systematically related to how people evaluate their bodies and to eating-related symptoms, in line with evidence that body dissatisfaction is associated with both BMI and depression (Morán et al., 2024).

Higher BMI was associated with lower body self-esteem and more eating disorder symptoms in both groups, and both mediators were independently associated with depressive symptoms. These findings are consistent with mediation studies that place body-related variables between BMI and emotional distress (Beltrán-Garrayo et al., 2024; Lin et al., 2025; Park and Ju, 2021) and with evidence that body image, depression and BMI are interrelated (Paans et al., 2018; Sharpe et al., 2018). The bootstrap intervals of the four specific indirect effects excluded zero, which supports the participation of both mediators in both groups. Even so, the indirect effects were small in standardized terms (total indirect *β* = 0.211 in women and 0.115 in men), so body self-esteem and eating disorder symptoms explain only a modest part of how BMI relates to depressive symptoms, and other mediators are likely to be involved.

The paths from BMI to the mediators differed between groups (Δ*χ^2^* (2) = 8.44, *p* = 0.015), and the path to eating disorder symptoms was stronger in women (difference in *b* = 0.521), in line with the greater emphasis placed on thinness in the sociocultural standards for women (Tiggemann, 2004). The indirect effects through eating disorder symptoms and in total were also larger in women. However, these contrasts were exploratory, did not remain significant after correction for multiple comparisons, and the contrast between the two pathways within each group did not differ between women and men (difference of contrasts = 0.009, *p* = 0.234). Therefore, the data do not support the second hypothesis in its full form: the eating disorder pathway was not stronger relative to the body self-esteem pathway in women, and the body self-esteem pathway was not stronger in men. This is consistent with evidence that the mediating role of body esteem does not differ between boys and girls (Beltrán-Garrayo et al., 2024). The most defensible reading is that the architecture of the relations is similar in women and men, with quantitative differences in the strength of the associations between BMI and the mediators, and not that different pathways operate in each group.

Although the cross-sectional design prevents causal conclusions, the results suggest that, among young adults with higher BMI, addressing body self-esteem and eating-related cognitions may be relevant for depressive symptoms, and that approaches focused only on weight may leave these processes unaddressed. Because evidence for gender-specific pathways is not robust, gender-sensitive adaptations should be considered tentative, although the stronger link between BMI and eating disorder symptoms in women suggests paying special attention to this dimension in women.

### Limitations and future directions

Several limitations should be acknowledged. First, recruitment relied on snowball sampling that began with psychology students at private universities. This strategy probably introduces selection effects, such as greater psychological literacy or particular distributions of BMI and body image, which limit generalization to other young adults in Colombia and Latin America. Second, weight and height were self-reported, and BMI was calculated from them. Self-reported anthropometrics contain error that may itself be related to body dissatisfaction and disordered eating, the key variables of this study, so the associations with BMI may be biased in an unknown direction. Measured anthropometry would avoid this problem. Third, underweight participants were excluded, so the study covers BMI from 18.5 kg/m^2^ upward and cannot evaluate the lower arm of a U-shaped association with depression, and it may not represent processes that operate at low BMI. In addition, six women with a BMI above 50 kg/m^2^ were excluded after influence diagnostics showed that they had a disproportionate influence on the estimated slopes. When they were included, the direct effect in women was no longer significant, the pattern became indirect-only mediation, and equality of paths across groups was not rejected. Since this decision was made after examining the data, results with and without these participants are reported in full in the Supplementary Materials, and the results should be generalized only to a BMI up to 50 kg/m^2^. Future research should use longitudinal designs, measured anthropometry, samples that include the full BMI range, models that allow nonlinear associations, and should incorporate anxiety and other mediators.

## Conclusion

In young Colombian adults aged 18–30 years, BMI showed no net association with depressive symptoms, but it was associated with lower body self-esteem and more eating disorder symptoms, which in turn were associated with more depressive symptoms. In men, this pattern corresponded to indirect-only mediation. In women, the positive indirect effect was offset by a negative direct association, which resulted in a null total effect. The associations between BMI and the mediators, especially eating disorder symptoms, were stronger in women, but the evidence does not support that the pathways themselves are distinct between women and men. These findings suggest that the relationship between weight and depressive symptoms cannot be understood through BMI alone and that body self-esteem and eating-related symptoms deserve attention in prevention and intervention efforts with young adults. However, the effects were small, the design was cross-sectional, and the results in women were sensitive to a small number of extreme BMI values, so they should be replicated in longitudinal designs with measured anthropometry.

## Data Availability

The datasets presented in this study can be found in online repositories. The names of the repository/repositories and accession number(s) can be found at: https://osf.io/jtqms/overview?view_only=13ab90c250d548bf95f4f2ca60a651b7.

## References

[ref1] American Psychiatric Association (2013). Diagnostic and Statistical Manual of Mental Disorders. 5th Edn. Washington, DC: American Psychiatric Publishing.

[ref2] Andres F. E. Thornborrow T. Bowie W. N. Chamorro A. M. De la Rosa G. Evans E. H. . (2023). Validation of a Latin-American Spanish version of the body esteem scale for adolescents and adults (BESAA-LA) in Colombian and Nicaraguan adults. J. Eat. Disord. 11:219. doi: 10.1186/s40337-023-00942-5 PMC10709846 38066645

[ref3] Belsley D. A. Kuh E. Welsch R. E. (1980). Regression Diagnostics: Identifying Influential Data and Sources of Collinearity. New York: Wiley.

[ref4] Beltrán-Garrayo L. Larsen J. K. Eisinga R. Vink J. M. Blanco M. Graell M. . (2024). Childhood obesity and adolescent follow-up depressive symptoms: exploring a moderated mediation model of body esteem and gender. Eur. Child Adolesc. Psychiatry 33, 2859–2869. doi: 10.1007/s00787-023-02348-9, 38326572 PMC11272700

[ref5] Blundell E. De Stavola B. L. Kellock M. D. Kelly Y. Lewis G. McMunn A. . (2024). Longitudinal pathways between childhood BMI, body dissatisfaction, and adolescent depression: an observational study using the UK millennium cohort study. Lancet Psychiatry 11, 47–55. doi: 10.1016/S2215-0366(23)00365-6, 38101872 PMC11139652

[ref6] Bonett D. G. Wright T. A. (2000). Sample size requirements for estimating Pearson, Kendall and spearman correlations. Psychometrika 65, 23–28. doi: 10.1007/BF02294183

[ref7] Cassiani-Miranda C. A. Cuadros-Cruz A. K. Torres-Pinzón H. Scoppetta O. Pinzón-Tarrazona J. H. López-Fuentes W. Y. . (2021). Validity of the patient health Questionnaire-9 (PHQ-9) for depression screening in adult primary care users in Bucaramanga, Colombia. Rev. Colomb. Psiquiatr. 50, 11–21. doi: 10.1016/j.rcpeng.2019.09.002, 33648690

[ref8] Chen F. F. (2007). Sensitivity of goodness of fit indexes to lack of measurement invariance. Struct. Equ. Model. 14, 464–504. doi: 10.1080/10705510701301834

[ref9] de Wit L. M. van Straten A. van Herten M. Penninx B. W. J. H. Cuijpers P. (2009). Depression and body mass index, a u-shaped association. BMC Public Health 9:14. doi: 10.1186/1471-2458-9-14, 19144098 PMC2631467

[ref10] Devoe D. J. Han A. Anderson A. Katzman D. K. Patten S. B. Soumbasis A. . (2023). The impact of the COVID-19 pandemic on eating disorders: a systematic review. Int. J. Eat. Disord. 56, 5–25. doi: 10.1002/eat.23704, 35384016 PMC9087369

[ref11] Fairburn C. G. Beglin S. J. (1994). Assessment of eating disorders: interview or self-report questionnaire? Int. J. Eat. Disord. 16, 363–370. doi: 10.1002/1098-108X(199412)16:4<>3.0.CO;2, 7866415

[ref12] Ferreiro F. Seoane G. Senra C. (2014). Toward understanding the role of body dissatisfaction in the gender differences in depressive symptoms and disordered eating: a longitudinal study during adolescence. J. Adolesc. 37, 73–84. doi: 10.1016/j.adolescence.2013.10.013, 24331307

[ref13] Foster L. Lundh L.-G. Daukantaitė D. (2023). Disordered eating in a 10-year perspective from adolescence to young adulthood: stability, change, and body dissatisfaction as a predictor. Scand. J. Psychol. 65, 32–41. doi: 10.1111/sjop.12950, 37491950

[ref14] Goldschmidt A. B. Wall M. M. Loth K. A. Neumark-Sztainer D. (2015). Risk factors for disordered eating in overweight adolescents and young adults. J. Pediatr. Psychol. 40, 1048–1055. doi: 10.1093/jpepsy/jsv053, 26050243 PMC4723677

[ref15] Griffiths S. Hay P. Mitchison D. Mond J. M. McLean S. A. Rodgers B. . (2016). Sex differences in the relationships between body dissatisfaction, quality of life and psychological distress. Aust. N. Z. J. Public Health 40, 518–522. doi: 10.1111/1753-6405.12538, 27372301

[ref16] Hambleton A. Pepin G. Le A. Maloney D. National Eating Disorder Research Consortium Touyz S. . (2022). Psychiatric and medical comorbidities of eating disorders: findings from a rapid review of the literature. J. Eat. Disord. 10:132. doi: 10.1186/s40337-022-00654-2, 36064606 PMC9442924

[ref17] Hoenig J. M. Heisey D. M. (2001). The abuse of power: the pervasive fallacy of power calculations for data analysis. Am. Stat. 55, 19–24. doi: 10.1198/000313001300339897

[ref18] Hudson J. I. Hiripi E. Pope H. G. Jr. Kessler R. C. (2007). The prevalence and correlates of eating disorders in the National Comorbidity Survey Replication. Biol. Psychiatry 61, 348–358. doi: 10.1016/j.biopsych.2006.03.040, 16815322 PMC1892232

[ref19] Kliem S. Mößle T. Zenger M. Strauß B. Brähler E. Hilbert A. (2016). The eating disorder examination-questionnaire 8: a brief measure of eating disorder psychopathology (EDE-Q8). Int. J. Eat. Disord. 49, 613–616. doi: 10.1002/eat.22487, 26711183

[ref20] Kolar D. R. Mebarak M. (2022). An update on the epidemiology of eating disorders in Latin America: current findings and future challenges. Curr. Opin. Psychiatry 35, 385–389. doi: 10.1097/YCO.0000000000000813, 35855501

[ref21] Kroenke K. Spitzer R. L. Williams J. B. W. (2001). The PHQ-9: validity of a brief depression severity measure. J. Gen. Intern. Med. 16, 606–613. doi: 10.1046/j.1525-1497.2001.016009606.x, 11556941 PMC1495268

[ref22] Lin J. F. Chuang S. P. Wu J. Y. W. (2025). The association between obesity and depression: the mediating roles of perceived overweight, body image dissatisfaction, and weight-based rejection sensitivity. J. Med. Sci. 45, 249–257. doi: 10.4103/jmedsci.jmedsci_84_25

[ref23] Luppino F. S. de Wit L. M. Bouvy P. F. Stijnen T. Cuijpers P. Penninx B. W. J. H. . (2010). Overweight, obesity, and depression: a systematic review and meta-analysis of longitudinal studies. Arch. Gen. Psychiatry 67, 220–229. doi: 10.1001/archgenpsychiatry.2010.2, 20194822

[ref24] Malik N. Ali N. (2021). The relationship with self-esteem, anxiety and depression controlling for body mass in adolescents and young adults with perceived body image dissatisfaction. Int. J. Indian Psychol. 9, 14–30. doi: 10.25215/0902.003

[ref25] Mendelson B. K. Mendelson M. J. White D. R. (2001). Body-esteem scale for adolescents and adults. J. Pers. Assess. 76, 90–106. doi: 10.1207/S15327752JPA7601_6, 11206302

[ref26] Morán C. Victoriano M. Parra J. Ibacache X. Pérez R. Sánchez J. . (2024). Body dissatisfaction, self-esteem, depressive symptoms, and nutritional status in adolescents. Andes pediatr. 95, 69–76. doi: 10.32641/andespediatr.v95i1.4779, 38587346

[ref27] Paans N. P. G. Bot M. Brouwer I. A. Visser M. Penninx B. W. J. H. (2018). Contributions of depression and body mass index to body image. J. Psychiatr. Res. 103, 18–25. doi: 10.1016/j.jpsychires.2018.05.003, 29758472

[ref28] Park H.-R. Ju H. O. (2021). Impact of body mass index on eating disorders in adolescents with type 1 diabetes: focusing on mediating effect of body dissatisfaction, self-esteem, and depression. J. Korean Soc. Matern. Child Health 25, 121–129. doi: 10.21896/jksmch.2021.25.2.121

[ref29] Preacher K. J. Hayes A. F. (2008). Asymptotic and resampling strategies for assessing and comparing indirect effects in multiple mediator models. Behav. Res. Methods 40, 879–891. doi: 10.3758/BRM.40.3.879, 18697684

[ref30] Quittkat H. L. Hartmann A. S. Düsing R. Buhlmann U. Vocks S. (2019). Body dissatisfaction, importance of appearance, and body appreciation in men and women over the lifespan. Front. Psych. 10:864. doi: 10.3389/fpsyt.2019.00864, 31920737 PMC6928134

[ref31] R Core Team (2025). R: A Language and Environment for Statistical Computing [Computer software]. Vienna: R Foundation for Statistical Computing.

[ref32] Racine N. McArthur B. A. Cooke J. E. Eirich R. Zhu J. Madigan S. (2021). Global prevalence of depressive and anxiety symptoms in children and adolescents during COVID-19: a meta-analysis. JAMA Pediatr. 175, 1142–1150. doi: 10.1001/jamapediatrics.2021.2482, 34369987 PMC8353576

[ref33] Rosseel Y. Jorgensen T. D. De Wilde L. (2025) Lavaan: Latent Variable Analysis (R package version 0.6-21) [Computer software]. Comprehensive R Archive Network (CRAN).

[ref34] Sadler G. R. Lee H.-C. Lim R. S.-H. Fullerton J. (2010). Recruitment of hard-to-reach population subgroups via adaptations of the snowball sampling strategy. Nurs. Health Sci. 12, 369–374. doi: 10.1111/j.1442-2018.2010.00541.x, 20727089 PMC3222300

[ref35] Sharpe H. Patalay P. Choo T.-H. Wall M. Mason S. M. Goldschmidt A. B. . (2018). Bidirectional associations between body dissatisfaction and depressive symptoms from adolescence through early adulthood. Dev. Psychopathol. 30, 1447–1458. doi: 10.1017/S0954579417001663, 29144209 PMC6343674

[ref36] Shorey S. Ng E. D. Wong C. H. J. (2022). Global prevalence of depression and elevated depressive symptoms among adolescents: a systematic review and meta-analysis. Br. J. Clin. Psychol. 61, 287–305. doi: 10.1111/bjc.12333, 34569066

[ref37] Tiggemann M. (2004). Body image across the adult life span: stability and change. Body Image 1, 29–41. doi: 10.1016/S1740-1445(03)00002-0, 18089139

[ref38] Tiggemann M. Lynch J. E. (2001). Body image across the life span in adult women: the role of self-objectification. Dev. Psychol. 37, 243–253. doi: 10.1037/0012-1649.37.2.243, 11269392

[ref39] Tomczak M. Tomczak E. (2014). The need to report effect size estimates revisited: an overview of some recommended measures of effect size. Trends Sport Sci. 21, 19–25.

[ref40] World Health Organization (2025) Mental Health of Adolescents [Fact Sheet]. Geneva, Switzerland: World Health Organization. Available online at: https://www.wbc.poznan.pl/publication/413565 (accessed September 21, 2026)

[ref41] Zhao X. Lynch J. G. Jr. Chen Q. (2010). Reconsidering baron and Kenny: myths and truths about mediation analysis. J. Consum. Res. 37, 197–206. doi: 10.1086/651257

