# Supplementary material for: Psychological pathways linking body mass index and depression in women and men in a Colombian sociodemographic context
Source: Front Psychol. 2026 Oct 29;17:1980836. doi: 10.3389/fpsyg.2026.1980836 (PMC13754868; doi:10.3389/fpsyg.2026.1980836)
Supplement: Supplementary file 1 [file Table_1.DOCX]

**Methodological Appendix (Supplementary Material)**

# 1. Overview and reproducibility

This appendix documents the analyses that support the article and reports every result that is not presented in the main text. All results were generated with a single R Markdown notebook that reads the processed data file and produces each table and figure of this appendix, so that the analyses can be reproduced from start to finish; the notebook is provided as a separate Supplementary File.

Analyses were run in R version 4.5.2 (R Core Team, 2025) with the lavaan package, version 0.6-21 (Rosseel, 2012), the semTools package, version 0.5-8, and the pwr package, version 1.3-0. Unless otherwise stated, structural models were estimated with robust maximum likelihood (MLR) and full information maximum likelihood (FIML), separately for women and men in a multigroup framework (group 1 = women, group 2 = men). Model fit is reported with the robust CFI, TLI and RMSEA and with the SRMR. Bootstrap confidence intervals used 5,000 resamples with a fixed seed (123).

# 2. Supplementary methods

## 2.1 Participants, exclusions and missing data

The processed data file contained 766 participants. Analyses were restricted to participants aged 18 to 30 years, and participants with underweight (BMI ≤ 18.5 kg/m²) and with a BMI above 50 kg/m² were excluded (Table S1). The exclusion of participants with a BMI above 50 kg/m² was decided after examining influence diagnostics (Section 2.5), and the results with and without these participants are compared in Tables S15 to S19. Missing data were handled with FIML in all structural models; the amount of missing data per variable is reported in Table S1.

## 2.2 Measurement models and reliability

Reliability was estimated with McDonald's omega from confirmatory factor analyses (CFA) of the final measurement models, fitted with MLR and FIML to the analytic sample and separately to women and men (Table S3). The BESAA-LA was modeled with three correlated factors (weight satisfaction, negative appearance and positive appearance) and a residual covariance between items 10 and 13. The EDE-Q8 was modeled with one factor and residual covariances between items 4 and 5 and between items 7 and 8. The PHQ-9 was modeled with one factor and a residual covariance between items 3 and 4. Cronbach's alpha and the average variance extracted (AVE) are reported for comparison.

## 2.3 Measurement invariance of the PHQ-9

The depression factor of the structural model (nine PHQ-9 indicators, without residual covariances) was tested for configural, metric and scalar invariance between women and men (Table S4). Following Chen (2007), for samples larger than 300, lack of invariance is indicated by a decrease in CFI of .010 or more together with an increase in RMSEA of .015 or more, or with an increase in SRMR larger than .030 for loadings and larger than .015 for intercepts. Because one of the groups has fewer than 300 participants and group sizes are unequal, a stricter reading is also shown (decrease in CFI of .005 or more together with an increase in RMSEA of .010 or more, or the same SRMR increases). Scaled chi-square difference tests are reported as complementary information because of their sensitivity to sample size. Score tests identify the equality constraints that contribute most to the misfit of the scalar model (Table S5).

## 2.4 Structural model, comparison between groups and bootstrap

BMI predicted body self-esteem and eating disorder symptoms (paths a1 and a2) and depressive symptoms (direct path c′); both mediators predicted depressive symptoms (paths b1 and b2) and were allowed to covary. Depression was a latent variable with nine indicators, and the mediators entered as standardized factor scores (M = 50, SD = 10). Specific indirect effects were the products a1 × b1 and a2 × b2, the total indirect effect was their sum, and the total effect was the sum of the direct and total indirect effects. Differences between women and men were computed as defined parameters of the multigroup model (delta method with robust standard errors), including the contrast between the two specific indirect effects within each group and the difference of these contrasts between groups (Table S9). Nested models in which paths were constrained to be equal across groups were compared with the freely estimated model using scaled chi-square difference tests (Table S10). Percentile bootstrap confidence intervals of the indirect and total effects were obtained with 5,000 resamples (Preacher & Hayes, 2008). The pattern of mediation was classified according to Zhao et al. (2010).

## 2.5 Sensitivity analyses

Four sets of sensitivity analyses were conducted. First, a residual covariance between PHQ-9 items 3 and 4 was added to the main model (Table S12). Second, a model was estimated in which depression, eating disorder symptoms and body self-esteem were latent variables, with body self-esteem represented as a second-order factor of the three BESAA-LA subscales; standardized coefficients are compared with those of the main model because the metrics of latent variables and factor scores differ (Table S13). Third, the structural model was re-estimated with equal loadings (metric) and with equal loadings and indicator intercepts (scalar); the intercepts of the two observed mediators were left free (Table S14).

Fourth, the influence of extreme BMI values was examined in the sample that includes them. For each group and outcome, linear regressions of the outcome on BMI were used to compute the leverage (hat value), Cook's distance and the DFBETAS of the BMI slope, and observations were flagged as influential by the influence.measures() function of the stats package on any of these criteria (Belsley et al., 1980) (Table S15 and Figure S1). Linearity was assessed by adding a quadratic term of centered BMI to these regressions (Table S16). The main model was then re-estimated under alternative BMI exclusion cutoffs of 40, 45, 50, 55 and 60 kg/m² and without exclusion (Table S17), in the sample including the extreme observations (Table S18) and with the natural logarithm of BMI as predictor (Table S19).

## 2.6 Sensitivity of the sample size

Instead of post hoc power calculated from observed effects, which is a function of the p-value and adds no information (Hoenig & Heisey, 2001), a sensitivity analysis reports the smallest effects that the achieved sample sizes could detect with 80% power at α = .05 (two-sided; Table S20). For indirect effects, power was approximated with the joint significance test (MacKinnon et al., 2002), whose power is the product of the power of the two paths that compose the effect.

# 3. List of supplementary tables and figures

| **Table S1** | Participant flow, exclusions and missing data |
| --- | --- |
| **Table S2** | Sample characteristics by group |
| **Table S3** | Reliability, average variance extracted and fit of the measurement models |
| **Table S4** | Measurement invariance of the PHQ-9 depression factor between women and men |
| **Table S5** | Score tests for the equality constraints of the scalar invariance model |
| **Table S6** | Pairwise comparisons between BMI categories |
| **Table S7** | Spearman and Pearson correlations with confidence intervals |
| **Table S8** | Main multigroup mediation model: fit, coefficients, mediation pattern and bootstrap intervals |
| **Table S9** | Wald tests of differences between women and men |
| **Table S10** | Nested-model comparisons across groups |
| **Table S11** | Total effect of BMI on depressive symptoms with and without mediators |
| **Table S12** | Sensitivity analysis: residual covariance between PHQ-9 items 3 and 4 |
| **Table S13** | Sensitivity analysis: fully latent model |
| **Table S14** | Sensitivity analysis: measurement constraints in the structural model |
| **Table S15** | Extreme BMI values: influence diagnostics |
| **Table S16** | Extreme BMI values: linearity |
| **Table S17** | Extreme BMI values: sensitivity to the exclusion cutoff |
| **Table S18** | Extreme BMI values: analyses including the extreme observations |
| **Table S19** | Extreme BMI values: log-transformed BMI |
| **Table S20** | Sensitivity analysis of the sample size |
| **Figure S1** | Body self-esteem, EDE-Q8 and depression scores by BMI |
| **Figure S2** | Direct, indirect and total effects of BMI by group |

# 4. Supplementary tables

## Table S1. Participant flow, exclusions and missing data

**Table S1a. Participant flow and exclusions**

| **Step** | **n** | **% of participants in the file** | **% of age-eligible participants (n = 713)** |
| --- | --- | --- | --- |
| Participants in the processed file (attention check passed) | 766 | 100.0 |  |
| Excluded: age > 30 years | 48 | 6.3 |  |
| Excluded: age not reported | 5 | 0.7 |  |
| Excluded: underweight (BMI ≤ 18.5) | 45 | 5.9 | 6.3 |
| Excluded: BMI not available | 0 | 0.0 | 0.0 |
| Excluded: BMI above 50 (extreme values) | 6 | 0.8 | 0.8 |
| Analytic sample | 662 | 86.4 |  |

*Note. The percentage of the last two exclusion steps is also shown relative to the participants who met the age criterion (n = 713).*

**Table S1b. Participants located exactly at a BMI cutpoint**

| **BMI cutpoint** | **n exactly at the cutpoint** |
| --- | --- |
| 18.5 | 0 |
| 25.0 | 0 |
| 30.0 | 0 |

*Note. BMI categories used intervals closed on the right (cutpoints 18.5, 25 and 30 kg/m²); no participant fell exactly on a cutpoint.*

**Table S1c. Missing data per variable in the analytic sample**

| **Variable** | **n missing** | **% missing** |
| --- | --- | --- |
| IMC | 0 | 0.00 |
| dsq1 | 23 | 3.47 |
| dsq2 | 23 | 3.47 |
| dsq3 | 23 | 3.47 |
| dsq4 | 23 | 3.47 |
| dsq5 | 23 | 3.47 |
| dsq6 | 23 | 3.47 |
| dsq7 | 23 | 3.47 |
| dsq8 | 23 | 3.47 |
| dsq9 | 23 | 3.47 |
| depresion | 23 | 3.47 |
| edeq | 0 | 0.00 |
| autoestima_corporal | 0 | 0.00 |

*Note. Missing values of the PHQ-9 items co-occurred: 23 participants (3.5%) did not answer any item, and 639 participants (96.5%) had complete data on all variables of the model. Missing data were handled with FIML.*

**Table S2. Sample characteristics, total and by group**

| **Variable** | **Total (N = 662)** | **Women (n = 398)** | **Men (n = 264)** |
| --- | --- | --- | --- |
| Age, M (SD) [min-max] | 21.75 (3.11) [18.0-30.0] | 21.52 (3.04) [18.0-30.0] | 22.11 (3.20) [18.0-30.0] |
| BMI, M (SD) [min-max] | 24.37 (4.08) [18.5-49.6] | 23.87 (3.90) [18.5-47.8] | 25.12 (4.25) [18.5-49.6] |
| Education, n (%): No universitario | 292 (44.1%) | 161 (40.5%) | 131 (49.6%) |
| Education, n (%): Universitario | 370 (55.9%) | 237 (59.5%) | 133 (50.4%) |
| Marital status, n (%): Comprometido | 315 (47.9%) | 201 (50.9%) | 114 (43.3%) |
| Marital status, n (%): Soltero | 343 (52.1%) | 194 (49.1%) | 149 (56.7%) |
| BMI category, n (%): Normal | 424 (64.0%) | 276 (69.3%) | 148 (56.1%) |
| BMI category, n (%): Sobrepeso | 181 (27.3%) | 94 (23.6%) | 87 (33.0%) |
| BMI category, n (%): Obesidad | 57 (8.6%) | 28 (7.0%) | 29 (11.0%) |

*Note. Age and BMI are presented as M (SD) [minimum-maximum]. Percentages of education and marital status are computed among participants with valid data.*

**Table S3. Reliability, average variance extracted and fit of the measurement models**

| **Scale** | **Factor** | **Sample** | **ω** | **α** | **AVE** | **CFI** | **TLI** | **RMSEA** | **SRMR** |
| --- | --- | --- | --- | --- | --- | --- | --- | --- | --- |
| BESAA (3 subscales) | Weight satisfaction | Total | 0.90 | 0.90 | 0.75 | 0.92 | 0.90 | 0.09 | 0.06 |
| BESAA (3 subscales) | Negative appearance | Total | 0.76 | 0.75 | 0.39 | 0.92 | 0.90 | 0.09 | 0.06 |
| BESAA (3 subscales) | Positive appearance | Total | 0.93 | 0.94 | 0.59 | 0.92 | 0.90 | 0.09 | 0.06 |
| BESAA (3 subscales) | Weight satisfaction | Women | 0.90 | 0.90 | 0.75 | 0.92 | 0.91 | 0.09 | 0.07 |
| BESAA (3 subscales) | Negative appearance | Women | 0.79 | 0.79 | 0.44 | 0.92 | 0.91 | 0.09 | 0.07 |
| BESAA (3 subscales) | Positive appearance | Women | 0.93 | 0.93 | 0.59 | 0.92 | 0.91 | 0.09 | 0.07 |
| BESAA (3 subscales) | Weight satisfaction | Men | 0.90 | 0.89 | 0.75 | 0.91 | 0.89 | 0.09 | 0.07 |
| BESAA (3 subscales) | Negative appearance | Men | 0.69 | 0.69 | 0.32 | 0.91 | 0.89 | 0.09 | 0.07 |
| BESAA (3 subscales) | Positive appearance | Men | 0.93 | 0.94 | 0.60 | 0.91 | 0.89 | 0.09 | 0.07 |
| EDE-Q8 | EDE-Q8 (single factor) | Total | 0.86 | 0.88 | 0.51 | 0.94 | 0.91 | 0.12 | 0.05 |
| EDE-Q8 | EDE-Q8 (single factor) | Women | 0.89 | 0.90 | 0.56 | 0.95 | 0.92 | 0.12 | 0.05 |
| EDE-Q8 | EDE-Q8 (single factor) | Men | 0.78 | 0.82 | 0.38 | 0.93 | 0.89 | 0.11 | 0.06 |
| PHQ-9 | PHQ-9 (single factor) | Total | 0.87 | 0.88 | 0.46 | 0.95 | 0.93 | 0.09 | 0.04 |
| PHQ-9 | PHQ-9 (single factor) | Women | 0.87 | 0.88 | 0.45 | 0.96 | 0.94 | 0.08 | 0.04 |
| PHQ-9 | PHQ-9 (single factor) | Men | 0.88 | 0.89 | 0.47 | 0.93 | 0.91 | 0.10 | 0.05 |

*Note. ω = McDonald's omega; α = Cronbach's alpha; AVE = average variance extracted. Fit indices are robust (CFI, TLI, RMSEA) and SRMR. In the total sample, omega ranged from .76 (BESAA negative appearance; AVE = .39) to .93 (BESAA positive appearance).*

**Table S4. Measurement invariance of the PHQ-9 depression factor between women and men (MLR, FIML)**

| **Model** | **χ²** | **df** | **CFI** | **TLI** | **RMSEA** | **SRMR** | **ΔCFI** | **ΔRMSEA** | **ΔSRMR** | **Δχ²** | **Δdf** | **p (Δχ²)** | **Decision** | **Decision (stricter criterion)** |
| --- | --- | --- | --- | --- | --- | --- | --- | --- | --- | --- | --- | --- | --- | --- |
| Configural | 183.69 | 54 | 0.929 | 0.905 | 0.101 | 0.046 |  |  |  |  |  |  |  |  |
| Metric | 195.99 | 62 | 0.927 | 0.915 | 0.095 | 0.057 | −0.002 | −0.005 | 0.010 | 11.63 | 8 | .169 | Supported | Supported |
| Scalar | 222.68 | 70 | 0.919 | 0.917 | 0.094 | 0.060 | −0.008 | −0.001 | 0.003 | 27.16 | 8 | < .001 | Supported | Supported |

*Note. Women, n = 398; men, n = 264. The decisions follow the criteria of Chen (2007) described in Section 2.3; ΔSRMR cutoffs were .030 (metric) and .015 (scalar). Δ values compare each model with the preceding one.*

**Table S5. Score tests for the equality constraints of the scalar invariance model (largest first)**

| **Constrained parameter (1)** | **Constrained parameter (2)** | **Chi-square** | **df** | **p** |
| --- | --- | --- | --- | --- |
| dsq5 ~1 (group 1) | dsq5 ~1 (group 2) | 13.27 | 1 | < .001 |
| dsq9 ~1 (group 1) | dsq9 ~1 (group 2) | 8.61 | 1 | .003 |
| dsq6 ~1 (group 1) | dsq6 ~1 (group 2) | 5.52 | 1 | .019 |
| depresion_ =~ dsq2 (group 1) | depresion_ =~ dsq2 (group 2) | 3.50 | 1 | .062 |
| depresion_ =~ dsq9 (group 1) | depresion_ =~ dsq9 (group 2) | 3.29 | 1 | .070 |
| depresion_ =~ dsq4 (group 1) | depresion_ =~ dsq4 (group 2) | 2.67 | 1 | .102 |
| dsq2 ~1 (group 1) | dsq2 ~1 (group 2) | 1.34 | 1 | .246 |
| depresion_ =~ dsq7 (group 1) | depresion_ =~ dsq7 (group 2) | 1.15 | 1 | .284 |

*Note. Group 1 = women; group 2 = men. Intercepts of items 5, 9 and 6 showed the largest values.*

**Table S6. Pairwise Wilcoxon rank-sum tests between BMI categories (Holm correction)**

| **Variable** | **Comparison** | **p (Holm)** |
| --- | --- | --- |
| Depression | Overweight vs Normal weight | 1.000 |
| Depression | Obesity vs Normal weight | 1.000 |
| Depression | Obesity vs Overweight | 1.000 |
| EDE-Q8 | Overweight vs Normal weight | < .001 |
| EDE-Q8 | Obesity vs Normal weight | < .001 |
| EDE-Q8 | Obesity vs Overweight | .147 |
| Body self-esteem | Overweight vs Normal weight | < .001 |
| Body self-esteem | Obesity vs Normal weight | < .001 |
| Body self-esteem | Obesity vs Overweight | < .001 |

*Note. n = 424 (normal weight), 181 (overweight) and 57 (obesity).*

**Table S7a. Spearman correlations with 95% confidence intervals**

| **Method** | **Sample** | **Pair** | **n** | **r** | **95% CI** | **p (Holm)** |
| --- | --- | --- | --- | --- | --- | --- |
| Spearman | Total | 1. BMI with 2. Depression | 639 | −.01 | [−.08, .07] | .867 |
| Spearman | Total | 1. BMI with 3. EDE-Q8 | 662 | .27 | [.20, .34] | < .001 |
| Spearman | Total | 1. BMI with 4. Body self-esteem | 662 | −.19 | [−.26, −.11] | < .001 |
| Spearman | Total | 2. Depression with 3. EDE-Q8 | 639 | .42 | [.35, .49] | < .001 |
| Spearman | Total | 2. Depression with 4. Body self-esteem | 639 | −.41 | [−.48, −.34] | < .001 |
| Spearman | Total | 3. EDE-Q8 with 4. Body self-esteem | 662 | −.55 | [−.60, −.49] | < .001 |
| Spearman | Women | 1. BMI with 2. Depression | 389 | .01 | [−.09, .11] | .846 |
| Spearman | Women | 1. BMI with 3. EDE-Q8 | 398 | .37 | [.28, .46] | < .001 |
| Spearman | Women | 1. BMI with 4. Body self-esteem | 398 | −.21 | [−.30, −.11] | < .001 |
| Spearman | Women | 2. Depression with 3. EDE-Q8 | 389 | .45 | [.36, .53] | < .001 |
| Spearman | Women | 2. Depression with 4. Body self-esteem | 389 | −.46 | [−.54, −.37] | < .001 |
| Spearman | Women | 3. EDE-Q8 with 4. Body self-esteem | 398 | −.59 | [−.66, −.52] | < .001 |
| Spearman | Men | 1. BMI with 2. Depression | 250 | .01 | [−.12, .13] | .888 |
| Spearman | Men | 1. BMI with 3. EDE-Q8 | 264 | .26 | [.14, .37] | < .001 |
| Spearman | Men | 1. BMI with 4. Body self-esteem | 264 | −.17 | [−.29, −.05] | .009 |
| Spearman | Men | 2. Depression with 3. EDE-Q8 | 250 | .31 | [.20, .42] | < .001 |
| Spearman | Men | 2. Depression with 4. Body self-esteem | 250 | −.33 | [−.44, −.21] | < .001 |
| Spearman | Men | 3. EDE-Q8 with 4. Body self-esteem | 264 | −.49 | [−.58, −.39] | < .001 |

*Note. Confidence intervals use Fisher's transformation with the standard error of Bonett and Wright (2000); p-values are Holm-adjusted within each set of six correlations. The number of observations varies across pairs because 23 participants (women, 9; men, 14) did not answer the PHQ-9.*

**Table S7b. Pearson correlations with 95% confidence intervals, total sample**

| **Method** | **Sample** | **Pair** | **n** | **r** | **95% CI** | **p (Holm)** |
| --- | --- | --- | --- | --- | --- | --- |
| Pearson | Total | 1. BMI with 2. Depression | 639 | .04 | [−.04, .12] | .328 |
| Pearson | Total | 1. BMI with 3. EDE-Q8 | 662 | .27 | [.19, .34] | < .001 |
| Pearson | Total | 1. BMI with 4. Body self-esteem | 662 | −.23 | [−.30, −.16] | < .001 |
| Pearson | Total | 2. Depression with 3. EDE-Q8 | 639 | .42 | [.35, .48] | < .001 |
| Pearson | Total | 2. Depression with 4. Body self-esteem | 639 | −.42 | [−.48, −.35] | < .001 |
| Pearson | Total | 3. EDE-Q8 with 4. Body self-esteem | 662 | −.55 | [−.61, −.50] | < .001 |

*Note. Presented for completeness; p-values are Holm-adjusted.*

## Table S8. Main multigroup mediation model

**Table S8a. Fit of the multigroup mediation model**

| **Chi-square (scaled)** | **df** | **CFI** | **TLI** | **RMSEA** | **RMSEA 90% CI** | **SRMR** | **n women** | **n men** |
| --- | --- | --- | --- | --- | --- | --- | --- | --- |
| 288.21 | 102 | 0.924 | 0.902 | 0.083 | [0.072, 0.095] | 0.048 | 398 | 264 |

**Table S8b. Women: unstandardized (b) and standardized (β) effects**

| **Effect** | **b** | **SE** | **z** | **p** | **95% CI** | **β (std.all)** |
| --- | --- | --- | --- | --- | --- | --- |
| BMI → Body self-esteem (a1) | −0.672 | 0.116 | −5.79 | < .001 | [−0.899, −0.444] | −0.265 |
| BMI → EDE-Q8 (a2) | 1.007 | 0.154 | 6.55 | < .001 | [0.706, 1.309] | 0.358 |
| Body self-esteem → Depression (b1) | −0.018 | 0.004 | −4.69 | < .001 | [−0.026, −0.011] | −0.300 |
| EDE-Q8 → Depression (b2) | 0.020 | 0.004 | 4.85 | < .001 | [0.012, 0.028] | 0.367 |
| BMI → Depression, direct (c’) | −0.028 | 0.008 | −3.51 | < .001 | [−0.044, −0.012] | −0.181 |
| Indirect via body self-esteem (a1*b1) | 0.012 | 0.003 | 3.63 | < .001 | [0.006, 0.019] | 0.080 |
| Indirect via EDE-Q8 (a2*b2) | 0.020 | 0.005 | 3.85 | < .001 | [0.010, 0.031] | 0.131 |
| Total indirect effect | 0.033 | 0.006 | 5.60 | < .001 | [0.021, 0.044] | 0.211 |
| Total effect (c’ + total indirect) | 0.005 | 0.009 | 0.53 | .597 | [−0.012, 0.021] | 0.029 |

*Note. Confidence intervals are based on robust standard errors (delta method for indirect and total effects).*

**Table S8c. Men: unstandardized (b) and standardized (β) effects**

| **Effect** | **b** | **SE** | **z** | **p** | **95% CI** | **β (std.all)** |
| --- | --- | --- | --- | --- | --- | --- |
| BMI → Body self-esteem (a1) | −0.493 | 0.153 | −3.21 | .001 | [−0.793, −0.192] | −0.207 |
| BMI → EDE-Q8 (a2) | 0.486 | 0.138 | 3.52 | < .001 | [0.215, 0.757] | 0.263 |
| Body self-esteem → Depression (b1) | −0.017 | 0.004 | −3.79 | < .001 | [−0.026, −0.008] | −0.304 |
| EDE-Q8 → Depression (b2) | 0.014 | 0.005 | 2.57 | .010 | [0.003, 0.025] | 0.196 |
| BMI → Depression, direct (c’) | −0.004 | 0.012 | −0.30 | .765 | [−0.027, 0.020] | −0.027 |
| Indirect via body self-esteem (a1*b1) | 0.008 | 0.003 | 2.46 | .014 | [0.002, 0.015] | 0.063 |
| Indirect via EDE-Q8 (a2*b2) | 0.007 | 0.004 | 1.89 | .059 | [−0.000, 0.014] | 0.052 |
| Total indirect effect | 0.015 | 0.005 | 2.78 | .005 | [0.004, 0.026] | 0.115 |
| Total effect (c’ + total indirect) | 0.012 | 0.010 | 1.15 | .249 | [−0.008, 0.031] | 0.087 |

*Note. Confidence intervals are based on robust standard errors (delta method for indirect and total effects).*

**Table S8d. Classification of the mediation pattern by group**

| **Group** | **Total indirect (b)** | **p indirect** | **Direct c’ (b)** | **p direct** | **Total effect (b)** | **p total** | **Classification (Zhao et al., 2010)** |
| --- | --- | --- | --- | --- | --- | --- | --- |
| Women | 0.033 | < .001 | −0.028 | < .001 | 0.005 | .597 | Competitive mediation; total effect not significant |
| Men | 0.015 | .005 | −0.004 | .765 | 0.012 | .249 | Indirect-only mediation; total effect not significant |

*Note. Classification according to Zhao et al. (2010): the pattern depends on the significance and sign of the indirect and direct effects and does not require a significant total effect.*

**Table S8e. Bootstrap percentile confidence intervals (5,000 resamples)**

| **Effect** | **Estimate** | **Bootstrap 95% CI (percentile)** |
| --- | --- | --- |
| Women: indirect via body self-esteem | 0.0123 | [0.0061, 0.0197] |
| Women: indirect via EDE-Q8 | 0.0203 | [0.0108, 0.0323] |
| Women: total indirect | 0.0326 | [0.0218, 0.0451] |
| Women: total effect | 0.0045 | [−0.0116, 0.0220] |
| Men: indirect via body self-esteem | 0.0083 | [0.0028, 0.0163] |
| Men: indirect via EDE-Q8 | 0.0068 | [0.0010, 0.0159] |
| Men: total indirect | 0.0151 | [0.0064, 0.0284] |
| Men: total effect | 0.0116 | [−0.0092, 0.0307] |
| Difference of the within-group contrasts (women minus men) | 0.0095 | [−0.0069, 0.0264] |

**Table S9. Wald tests of differences between women and men (delta method, robust standard errors)**

| **Contrast** | **Estimate** | **SE** | **z** | **p** | **95% CI** |
| --- | --- | --- | --- | --- | --- |
| BMI → Body self-esteem (a1): women minus men | −0.179 | 0.192 | −0.93 | .352 | [−0.556, 0.198] |
| BMI → EDE-Q8 (a2): women minus men | 0.521 | 0.207 | 2.52 | .012 | [0.116, 0.926] |
| Body self-esteem → Depression (b1): women minus men | −0.001 | 0.006 | −0.23 | .815 | [−0.013, 0.010] |
| EDE-Q8 → Depression (b2): women minus men | 0.006 | 0.007 | 0.89 | .374 | [−0.007, 0.020] |
| Direct effect (c’): women minus men | −0.024 | 0.014 | −1.70 | .090 | [−0.053, 0.004] |
| Indirect via body self-esteem: women minus men | 0.004 | 0.005 | 0.83 | .409 | [−0.005, 0.013] |
| Indirect via EDE-Q8: women minus men | 0.013 | 0.006 | 2.11 | .035 | [0.001, 0.026] |
| Total indirect effect: women minus men | 0.017 | 0.008 | 2.18 | .029 | [0.002, 0.033] |
| Total effect: women minus men | −0.007 | 0.013 | −0.53 | .593 | [−0.033, 0.019] |
| Within women: indirect via EDE-Q8 minus via body self-esteem | 0.008 | 0.007 | 1.20 | .231 | [−0.005, 0.021] |
| Within men: indirect via EDE-Q8 minus via body self-esteem | −0.002 | 0.004 | −0.34 | .731 | [−0.010, 0.007] |
| Difference of the within-group contrasts (women minus men) | 0.009 | 0.008 | 1.19 | .234 | [−0.006, 0.025] |

*Note. Differences are women minus men, computed as defined parameters of the multigroup model. The last row tests whether the difference between the indirect effect via EDE-Q8 and via body self-esteem differs between groups. The p-values are unadjusted; with Holm correction across the nine principal contrasts (first nine rows), the smallest adjusted p-value was .108.*

**Table S10. Nested-model comparisons: equality of structural paths across groups (each model versus the free model)**

| **Restriction** | **Scaled chi-square difference** | **df difference** | **p** | **Delta CFI** |
| --- | --- | --- | --- | --- |
| Paths a1 and a2 equal across groups | 8.44 | 2 | .015 | −0.002 |
| Paths b1 and b2 equal across groups | 1.29 | 2 | .525 | 0.000 |
| Direct path c’ equal across groups | 3.14 | 1 | .076 | −0.001 |
| All structural paths equal across groups | 12.34 | 5 | .030 | −0.003 |

*Note. Scaled chi-square difference tests. ΔCFI is the CFI of the constrained model minus the CFI of the free model.*

**Table S11. Total effect of BMI on depressive symptoms: mediation model versus model without mediators**

| **Group** | **Total effect, mediation model (b)** | **p (mediation model)** | **Total effect, model without mediators (b)** | **p (model without mediators)** | **95% CI (model without mediators)** | **β (std.all)** |
| --- | --- | --- | --- | --- | --- | --- |
| Women | 0.005 | .597 | 0.005 | .544 | [−0.012, 0.023] | 0.034 |
| Men | 0.012 | .249 | 0.011 | .283 | [−0.009, 0.030] | 0.082 |

*Note. In the mediation model the total effect is the sum of the direct and total indirect effects; in the other model, the latent depression factor is regressed only on BMI.*

**Table S12a. Fit with and without the residual covariance between PHQ-9 items 3 and 4**

| **Model** | **Chi-square** | **df** | **CFI** | **TLI** | **RMSEA** | **SRMR** |
| --- | --- | --- | --- | --- | --- | --- |
| Main model | 288.21 | 102 | 0.924 | 0.902 | 0.083 | 0.048 |
| With dsq3~~dsq4 | 246.03 | 100 | 0.941 | 0.922 | 0.074 | 0.045 |

**Table S12b. Key estimates with and without the residual covariance**

| **Group** | **Effect** | **Main model: b** | **With dsq3~~dsq4: b** | **Main model: p** | **With dsq3~~dsq4: p** | **Main model: β** | **With dsq3~~dsq4: β** |
| --- | --- | --- | --- | --- | --- | --- | --- |
| Women | a1 (BMI → body self-esteem) | −0.672 | −0.672 | < .001 | < .001 | −0.265 | −0.265 |
| Women | a2 (BMI → EDE-Q8) | 1.007 | 1.007 | < .001 | < .001 | 0.358 | 0.358 |
| Women | b1 (body self-esteem → depression) | −0.018 | −0.018 | < .001 | < .001 | −0.300 | −0.302 |
| Women | b2 (EDE-Q8 → depression) | 0.020 | 0.020 | < .001 | < .001 | 0.367 | 0.367 |
| Women | c′ (direct effect) | −0.028 | −0.029 | < .001 | < .001 | −0.181 | −0.186 |
| Women | Indirect via body self-esteem | 0.012 | 0.012 | < .001 | < .001 | 0.080 | 0.080 |
| Women | Indirect via EDE-Q8 | 0.020 | 0.020 | < .001 | < .001 | 0.131 | 0.131 |
| Women | Total indirect effect | 0.033 | 0.032 | < .001 | < .001 | 0.211 | 0.211 |
| Women | Total effect | 0.005 | 0.004 | .597 | .651 | 0.029 | 0.025 |
| Men | a1 (BMI → body self-esteem) | −0.493 | −0.493 | .001 | .001 | −0.207 | −0.207 |
| Men | a2 (BMI → EDE-Q8) | 0.486 | 0.486 | < .001 | < .001 | 0.263 | 0.263 |
| Men | b1 (body self-esteem → depression) | −0.017 | −0.017 | < .001 | < .001 | −0.304 | −0.302 |
| Men | b2 (EDE-Q8 → depression) | 0.014 | 0.014 | .010 | .009 | 0.196 | 0.200 |
| Men | c′ (direct effect) | −0.004 | −0.003 | .765 | .792 | −0.027 | −0.024 |
| Men | Indirect via body self-esteem | 0.008 | 0.008 | .014 | .015 | 0.063 | 0.063 |
| Men | Indirect via EDE-Q8 | 0.007 | 0.007 | .059 | .057 | 0.052 | 0.052 |
| Men | Total indirect effect | 0.015 | 0.015 | .005 | .006 | 0.115 | 0.115 |
| Men | Total effect | 0.012 | 0.012 | .249 | .229 | 0.087 | 0.091 |

*Note. b = unstandardized estimate; β = fully standardized estimate.*

**Table S13a. Fit of the fully latent multigroup model**

| **Model** | **Chi-square** | **df** | **CFI** | **TLI** | **RMSEA** | **SRMR** |
| --- | --- | --- | --- | --- | --- | --- |
| Fully latent model | 2768.36 | 1166 | 0.879 | 0.870 | 0.068 | 0.075 |

*Note. Depression, eating disorder symptoms and body self-esteem were latent variables; body self-esteem was specified as a second-order factor of the three BESAA-LA subscales. The model converged.*

**Table S13b. Standardized effects: main model (factor scores) versus fully latent model**

| **Group** | **Effect** | **Main model: β** | **Main model: p** | **Latent model: β** | **Latent model: p** |
| --- | --- | --- | --- | --- | --- |
| Women | a1 (BMI → body self-esteem) | −0.265 | < .001 | −0.344 | < .001 |
| Women | a2 (BMI → EDE-Q8) | 0.358 | < .001 | 0.390 | < .001 |
| Women | b1 (body self-esteem → depression) | −0.300 | < .001 | −0.377 | .011 |
| Women | b2 (EDE-Q8 → depression) | 0.367 | < .001 | 0.279 | .079 |
| Women | c′ (direct effect) | −0.181 | < .001 | −0.208 | < .001 |
| Women | Indirect via body self-esteem | 0.080 | < .001 | 0.130 | .012 |
| Women | Indirect via EDE-Q8 | 0.131 | < .001 | 0.109 | .068 |
| Women | Total indirect effect | 0.211 | < .001 | 0.239 | < .001 |
| Women | Total effect | 0.029 | .597 | 0.031 | .578 |
| Men | a1 (BMI → body self-esteem) | −0.207 | .001 | −0.257 | < .001 |
| Men | a2 (BMI → EDE-Q8) | 0.263 | < .001 | 0.302 | .105 |
| Men | b1 (body self-esteem → depression) | −0.304 | < .001 | −0.389 | .010 |
| Men | b2 (EDE-Q8 → depression) | 0.196 | .010 | 0.117 | .478 |
| Men | c′ (direct effect) | −0.027 | .765 | −0.049 | .592 |
| Men | Indirect via body self-esteem | 0.063 | .014 | 0.100 | .020 |
| Men | Indirect via EDE-Q8 | 0.052 | .059 | 0.035 | .456 |
| Men | Total indirect effect | 0.115 | .005 | 0.135 | .003 |
| Men | Total effect | 0.087 | .249 | 0.087 | .252 |

**Table S14a. Fit of the structural model with equality constraints on the measurement model**

| **Model** | **Chi-square** | **df** | **CFI** | **TLI** | **RMSEA** | **SRMR** |
| --- | --- | --- | --- | --- | --- | --- |
| Free loadings (main) | 288.21 | 102 | 0.924 | 0.902 | 0.083 | 0.048 |
| Metric | 298.66 | 110 | 0.923 | 0.908 | 0.081 | 0.055 |
| Scalar | 324.97 | 118 | 0.917 | 0.907 | 0.081 | 0.057 |

*Note. Metric: equal loadings across groups. Scalar: equal loadings and indicator intercepts; the intercepts of the two observed mediators were left free.*

**Table S14b. Key estimates under increasing measurement constraints**

| **Group** | **Effect** | **Free loadings (main): b** | **Metric: b** | **Scalar: b** | **Free loadings (main): p** | **Metric: p** | **Scalar: p** | **Free loadings (main): β** | **Metric: β** | **Scalar: β** |
| --- | --- | --- | --- | --- | --- | --- | --- | --- | --- | --- |
| Women | a1 (BMI → body self-esteem) | −0.672 | −0.672 | −0.672 | < .001 | < .001 | < .001 | −0.265 | −0.265 | −0.265 |
| Women | a2 (BMI → EDE-Q8) | 1.007 | 1.007 | 1.007 | < .001 | < .001 | < .001 | 0.358 | 0.358 | 0.358 |
| Women | b1 (body self-esteem → depression) | −0.018 | −0.018 | −0.018 | < .001 | < .001 | < .001 | −0.300 | −0.302 | −0.301 |
| Women | b2 (EDE-Q8 → depression) | 0.020 | 0.019 | 0.020 | < .001 | < .001 | < .001 | 0.367 | 0.366 | 0.366 |
| Women | c′ (direct effect) | −0.028 | −0.027 | −0.027 | < .001 | < .001 | < .001 | −0.181 | −0.183 | −0.182 |
| Women | Indirect via body self-esteem | 0.012 | 0.012 | 0.012 | < .001 | < .001 | < .001 | 0.080 | 0.080 | 0.080 |
| Women | Indirect via EDE-Q8 | 0.020 | 0.020 | 0.020 | < .001 | < .001 | < .001 | 0.131 | 0.131 | 0.131 |
| Women | Total indirect effect | 0.033 | 0.031 | 0.032 | < .001 | < .001 | < .001 | 0.211 | 0.211 | 0.211 |
| Women | Total effect | 0.005 | 0.004 | 0.004 | .597 | .614 | .609 | 0.029 | 0.028 | 0.029 |
| Men | a1 (BMI → body self-esteem) | −0.493 | −0.493 | −0.493 | .001 | .001 | .001 | −0.207 | −0.207 | −0.207 |
| Men | a2 (BMI → EDE-Q8) | 0.486 | 0.486 | 0.486 | < .001 | < .001 | < .001 | 0.263 | 0.263 | 0.263 |
| Men | b1 (body self-esteem → depression) | −0.017 | −0.018 | −0.018 | < .001 | < .001 | < .001 | −0.304 | −0.306 | −0.306 |
| Men | b2 (EDE-Q8 → depression) | 0.014 | 0.015 | 0.015 | .010 | .009 | .009 | 0.196 | 0.197 | 0.196 |
| Men | c′ (direct effect) | −0.004 | −0.004 | −0.004 | .765 | .746 | .740 | −0.027 | −0.029 | −0.030 |
| Men | Indirect via body self-esteem | 0.008 | 0.009 | 0.009 | .014 | .011 | .011 | 0.063 | 0.063 | 0.064 |
| Men | Indirect via EDE-Q8 | 0.007 | 0.007 | 0.007 | .059 | .053 | .053 | 0.052 | 0.052 | 0.052 |
| Men | Total indirect effect | 0.015 | 0.016 | 0.016 | .005 | .004 | .004 | 0.115 | 0.115 | 0.115 |
| Men | Total effect | 0.012 | 0.012 | 0.012 | .249 | .254 | .259 | 0.087 | 0.086 | 0.085 |

*Note. b = unstandardized estimate; β = fully standardized estimate.*

**Table S15a. Highest BMI values in the sample that includes the extreme observations**

| **Group** | **BMI** | **z score** | **In analytic sample** |
| --- | --- | --- | --- |
| Women | 66.4 | 7.8 | no |
| Women | 65.3 | 7.6 | no |
| Women | 62.7 | 7.1 | no |
| Women | 59.9 | 6.6 | no |
| Women | 58.1 | 6.2 | no |
| Women | 52.9 | 5.3 | no |
| Men | 49.6 | 4.6 | yes |
| Women | 47.8 | 4.3 | yes |
| Men | 40.2 | 2.9 | yes |
| Men | 38.9 | 2.6 | yes |
| Men | 38.3 | 2.5 | yes |
| Women | 37.7 | 2.4 | yes |

*Note. The z score is computed with the mean and standard deviation of BMI in the sample that includes the extreme observations. 'In analytic sample' indicates whether the participant was retained (BMI ≤ 50 kg/m²).*

**Table S15b. Influential observations and BMI slope with all observations and in the analytic sample**

| **Group** | **Outcome** | **n observations** | **n flagged as influential** | **of which extreme** | **BMI slope, all observations** | **BMI slope, analytic sample** |
| --- | --- | --- | --- | --- | --- | --- |
| Men | Body self-esteem | 264 | 8 | 0 | −0.493 | −0.493 |
| Men | Depression | 250 | 8 | 0 | 0.194 | 0.194 |
| Men | EDE-Q8 | 264 | 8 | 0 | 0.486 | 0.486 |
| Women | Body self-esteem | 404 | 7 | 6 | −0.204 | −0.672 |
| Women | Depression | 394 | 7 | 5 | −0.010 | 0.079 |
| Women | EDE-Q8 | 404 | 7 | 6 | 0.350 | 1.007 |

*Note. Regressions of each outcome on BMI within each group. Observations were flagged as influential by influence.measures() on leverage, Cook's distance or DFBETAS of the BMI slope (Belsley et al., 1980).*

**Table S15c. Influence diagnostics of the extreme observations in the regressions of the mediators on BMI**

| **Group** | **Outcome** | **BMI** | **Leverage** | **Cook’s D** | **DFBETAS (BMI slope)** | **Flagged** |
| --- | --- | --- | --- | --- | --- | --- |
| Women | Body self-esteem | 66.4 | 0.126 | 0.020 | 0.20 | yes |
| Women | Body self-esteem | 65.3 | 0.119 | 0.010 | 0.14 | yes |
| Women | Body self-esteem | 62.7 | 0.105 | 0.268 | 0.73 | yes |
| Women | Body self-esteem | 59.9 | 0.091 | 0.213 | 0.65 | yes |
| Women | Body self-esteem | 58.1 | 0.082 | 0.041 | 0.28 | yes |
| Women | Body self-esteem | 52.9 | 0.059 | 0.195 | 0.62 | yes |
| Women | EDE-Q8 | 66.4 | 0.126 | 0.231 | −0.68 | yes |
| Women | EDE-Q8 | 65.3 | 0.119 | 0.000 | −0.01 | yes |
| Women | EDE-Q8 | 62.7 | 0.105 | 0.347 | −0.83 | yes |
| Women | EDE-Q8 | 59.9 | 0.091 | 0.279 | −0.74 | yes |
| Women | EDE-Q8 | 58.1 | 0.082 | 0.175 | −0.58 | yes |
| Women | EDE-Q8 | 52.9 | 0.059 | 0.145 | −0.53 | yes |

*Note. Leverage = hat value; DFBETAS refers to the BMI slope.*

**Figure S1. Body self-esteem, EDE-Q8 and depression scores by BMI in women and men**

*Note. Gray points: participants in the analytic sample. Red points: participants with a BMI above 50 kg/m², excluded from the analytic sample. Solid black line: linear fit. Dashed blue line: LOESS smoother. Scores are standardized (M = 50, SD = 10).*

**Table S16. Quadratic term of BMI in the regression of each outcome on BMI**

| **Group** | **Outcome** | **Quadratic term, all observations (b)** | **p, all observations** | **Quadratic term, analytic sample (b)** | **p, analytic sample** |
| --- | --- | --- | --- | --- | --- |
| Women | Body self-esteem | 0.0195 | < .001 | −0.0121 | .452 |
| Women | EDE-Q8 | −0.0347 | < .001 | −0.0563 | < .001 |
| Women | Depression | −0.0033 | .545 | −0.0032 | .847 |
| Men | Body self-esteem | −0.0070 | .667 | −0.0070 | .667 |
| Men | EDE-Q8 | −0.0282 | .023 | −0.0282 | .023 |
| Men | Depression | 0.0295 | .081 | 0.0295 | .081 |

*Note. b is the coefficient of squared centered BMI in the linear regression of each outcome within each group, with all observations and in the analytic sample.*

**Table S17. Main model under alternative BMI exclusion cutoffs: standardized coefficients (p-values) and mediation pattern**

| **BMI cutoff** | **n excluded (women / men)** | **Women: a1** | **Women: a2** | **Women: c’** | **Women: total indirect** | **Women: total effect** | **Women: pattern** | **Men: pattern** |
| --- | --- | --- | --- | --- | --- | --- | --- | --- |
| ≤ 40 | 7 / 2 | −0.262 (< .001) | 0.376 (< .001) | −0.182 (< .001) | 0.218 (< .001) | 0.036 (.540) | Competitive mediation | Indirect-only mediation |
| ≤ 45 | 7 / 1 | −0.262 (< .001) | 0.376 (< .001) | −0.182 (< .001) | 0.218 (< .001) | 0.036 (.540) | Competitive mediation | Indirect-only mediation |
| ≤ 50 | 6 / 0 | −0.265 (< .001) | 0.358 (< .001) | −0.181 (< .001) | 0.211 (< .001) | 0.029 (.597) | Competitive mediation | Indirect-only mediation |
| ≤ 55 | 5 / 0 | −0.215 (.003) | 0.315 (< .001) | −0.167 (.001) | 0.176 (< .001) | 0.010 (.862) | Competitive mediation | Indirect-only mediation |
| ≤ 60 | 3 / 0 | −0.158 (.036) | 0.236 (.006) | −0.143 (.003) | 0.127 (.019) | −0.016 (.744) | Competitive mediation | Indirect-only mediation |
| None (all participants) | 0 / 0 | −0.122 (.053) | 0.190 (.010) | −0.108 (.076) | 0.098 (.024) | −0.010 (.859) | Indirect-only mediation | Indirect-only mediation |

*Note. Standardized coefficients (β) with p-values in parentheses for women. n excluded refers to participants with a BMI above the cutoff; the last row applies no exclusion. The mediation pattern is classified following Zhao et al. (2010).*

## Table S18. Analyses including the extreme observations

**Table S18a. Model fit in the analytic sample and including the extreme observations**

| **Model** | **Chi-square** | **df** | **CFI** | **TLI** | **RMSEA** | **SRMR** |
| --- | --- | --- | --- | --- | --- | --- |
| Analytic sample | 288.21 | 102 | 0.924 | 0.902 | 0.083 | 0.048 |
| Including BMI > 50 | 280.43 | 102 | 0.926 | 0.905 | 0.081 | 0.046 |

*Note. The sample including the extreme observations comprises 404 women and 264 men.*

**Table S18b. Key estimates in the analytic sample and including the extreme observations**

| **Group** | **Effect** | **Analytic sample: b** | **Including BMI > 50: b** | **Analytic sample: p** | **Including BMI > 50: p** | **Analytic sample: β** | **Including BMI > 50: β** |
| --- | --- | --- | --- | --- | --- | --- | --- |
| Women | a1 (BMI → body self-esteem) | −0.672 | −0.204 | < .001 | .053 | −0.265 | −0.122 |
| Women | a2 (BMI → EDE-Q8) | 1.007 | 0.350 | < .001 | .010 | 0.358 | 0.190 |
| Women | b1 (body self-esteem → depression) | −0.018 | −0.018 | < .001 | < .001 | −0.300 | −0.293 |
| Women | b2 (EDE-Q8 → depression) | 0.020 | 0.018 | < .001 | < .001 | 0.367 | 0.327 |
| Women | c′ (direct effect) | −0.028 | −0.011 | < .001 | .076 | −0.181 | −0.108 |
| Women | Indirect via body self-esteem | 0.012 | 0.004 | < .001 | .075 | 0.080 | 0.036 |
| Women | Indirect via EDE-Q8 | 0.020 | 0.006 | < .001 | .029 | 0.131 | 0.062 |
| Women | Total indirect effect | 0.033 | 0.010 | < .001 | .024 | 0.211 | 0.098 |
| Women | Total effect | 0.005 | −0.001 | .597 | .859 | 0.029 | −0.010 |
| Men | a1 (BMI → body self-esteem) | −0.493 | −0.493 | .001 | .001 | −0.207 | −0.207 |
| Men | a2 (BMI → EDE-Q8) | 0.486 | 0.486 | < .001 | < .001 | 0.263 | 0.263 |
| Men | b1 (body self-esteem → depression) | −0.017 | −0.017 | < .001 | < .001 | −0.304 | −0.304 |
| Men | b2 (EDE-Q8 → depression) | 0.014 | 0.014 | .010 | .010 | 0.196 | 0.196 |
| Men | c′ (direct effect) | −0.004 | −0.004 | .765 | .765 | −0.027 | −0.027 |
| Men | Indirect via body self-esteem | 0.008 | 0.008 | .014 | .014 | 0.063 | 0.063 |
| Men | Indirect via EDE-Q8 | 0.007 | 0.007 | .059 | .059 | 0.052 | 0.052 |
| Men | Total indirect effect | 0.015 | 0.015 | .005 | .005 | 0.115 | 0.115 |
| Men | Total effect | 0.012 | 0.012 | .249 | .249 | 0.087 | 0.087 |

*Note. b = unstandardized estimate; β = fully standardized estimate.*

**Table S18c. Women including the extreme observations: unstandardized (b) and standardized (β) effects**

| **Effect** | **b** | **SE** | **z** | **p** | **95% CI** | **β (std.all)** |
| --- | --- | --- | --- | --- | --- | --- |
| BMI → Body self-esteem (a1) | −0.204 | 0.105 | −1.94 | .053 | [−0.410, 0.002] | −0.122 |
| BMI → EDE-Q8 (a2) | 0.350 | 0.136 | 2.58 | .010 | [0.084, 0.616] | 0.190 |
| Body self-esteem → Depression (b1) | −0.018 | 0.004 | −4.54 | < .001 | [−0.025, −0.010] | −0.293 |
| EDE-Q8 → Depression (b2) | 0.018 | 0.004 | 4.43 | < .001 | [0.010, 0.026] | 0.327 |
| BMI → Depression, direct (c’) | −0.011 | 0.006 | −1.78 | .076 | [−0.023, 0.001] | −0.108 |
| Indirect via body self-esteem (a1*b1) | 0.004 | 0.002 | 1.78 | .075 | [−0.000, 0.008] | 0.036 |
| Indirect via EDE-Q8 (a2*b2) | 0.006 | 0.003 | 2.18 | .029 | [0.001, 0.012] | 0.062 |
| Total indirect effect | 0.010 | 0.004 | 2.26 | .024 | [0.001, 0.018] | 0.098 |
| Total effect (c’ + total indirect) | −0.001 | 0.006 | −0.18 | .859 | [−0.013, 0.010] | −0.010 |

**Table S18d. Men including the extreme observations: unstandardized (b) and standardized (β) effects**

| **Effect** | **b** | **SE** | **z** | **p** | **95% CI** | **β (std.all)** |
| --- | --- | --- | --- | --- | --- | --- |
| BMI → Body self-esteem (a1) | −0.493 | 0.153 | −3.21 | .001 | [−0.793, −0.192] | −0.207 |
| BMI → EDE-Q8 (a2) | 0.486 | 0.138 | 3.52 | < .001 | [0.215, 0.757] | 0.263 |
| Body self-esteem → Depression (b1) | −0.017 | 0.004 | −3.79 | < .001 | [−0.026, −0.008] | −0.304 |
| EDE-Q8 → Depression (b2) | 0.014 | 0.005 | 2.57 | .010 | [0.003, 0.025] | 0.196 |
| BMI → Depression, direct (c’) | −0.004 | 0.012 | −0.30 | .765 | [−0.027, 0.020] | −0.027 |
| Indirect via body self-esteem (a1*b1) | 0.008 | 0.003 | 2.46 | .014 | [0.002, 0.015] | 0.063 |
| Indirect via EDE-Q8 (a2*b2) | 0.007 | 0.004 | 1.89 | .059 | [−0.000, 0.014] | 0.052 |
| Total indirect effect | 0.015 | 0.005 | 2.78 | .005 | [0.004, 0.026] | 0.115 |
| Total effect (c’ + total indirect) | 0.012 | 0.010 | 1.15 | .249 | [−0.008, 0.031] | 0.087 |

*Note. No man exceeded 50 kg/m², so the estimates for men are identical to those of the analytic sample.*

**Table S18e. Classification of the mediation pattern including the extreme observations**

| **Group** | **Total indirect (b)** | **p indirect** | **Direct c’ (b)** | **p direct** | **Total effect (b)** | **p total** | **Classification (Zhao et al., 2010)** |
| --- | --- | --- | --- | --- | --- | --- | --- |
| Women | 0.010 | .024 | −0.011 | .076 | −0.001 | .859 | Indirect-only mediation; total effect not significant |
| Men | 0.015 | .005 | −0.004 | .765 | 0.012 | .249 | Indirect-only mediation; total effect not significant |

**Table S18f. Wald tests of differences between women and men including the extreme observations**

| **Contrast** | **Estimate** | **SE** | **z** | **p** | **95% CI** |
| --- | --- | --- | --- | --- | --- |
| BMI → Body self-esteem (a1): women minus men | 0.289 | 0.186 | 1.55 | .120 | [−0.076, 0.653] |
| BMI → EDE-Q8 (a2): women minus men | −0.136 | 0.194 | −0.70 | .483 | [−0.515, 0.244] |
| Body self-esteem → Depression (b1): women minus men | −0.001 | 0.006 | −0.14 | .886 | [−0.012, 0.011] |
| EDE-Q8 → Depression (b2): women minus men | 0.004 | 0.007 | 0.56 | .574 | [−0.009, 0.017] |
| Direct effect (c’): women minus men | −0.007 | 0.013 | −0.54 | .587 | [−0.034, 0.019] |
| Indirect via body self-esteem: women minus men | −0.005 | 0.004 | −1.19 | .233 | [−0.012, 0.003] |
| Indirect via EDE-Q8: women minus men | −0.001 | 0.005 | −0.12 | .902 | [−0.010, 0.008] |
| Total indirect effect: women minus men | −0.005 | 0.007 | −0.76 | .449 | [−0.019, 0.008] |
| Total effect: women minus men | −0.013 | 0.012 | −1.09 | .277 | [−0.035, 0.010] |
| Within women: indirect via EDE-Q8 minus via body self-esteem | 0.003 | 0.002 | 1.10 | .272 | [−0.002, 0.007] |
| Within men: indirect via EDE-Q8 minus via body self-esteem | −0.002 | 0.004 | −0.34 | .731 | [−0.010, 0.007] |
| Difference of the within-group contrasts (women minus men) | 0.004 | 0.005 | 0.83 | .408 | [−0.006, 0.014] |

**Table S18g. Nested-model comparisons including the extreme observations**

| **Restriction** | **Scaled chi-square difference** | **df difference** | **p** | **Delta CFI** |
| --- | --- | --- | --- | --- |
| Paths a1 and a2 equal across groups | 2.39 | 2 | .303 | −0.000 |
| Paths b1 and b2 equal across groups | 0.51 | 2 | .776 | 0.001 |
| Direct path c’ equal across groups | 0.29 | 1 | .593 | 0.000 |
| All structural paths equal across groups | 2.94 | 5 | .709 | 0.001 |

**Table S18h. Bootstrap percentile confidence intervals including the extreme observations (5,000 resamples)**

| **Effect** | **Estimate** | **Bootstrap 95% CI (percentile)** |
| --- | --- | --- |
| Women: indirect via body self-esteem | 0.0036 | [0.0004, 0.0094] |
| Women: indirect via EDE-Q8 | 0.0062 | [0.0021, 0.0152] |
| Women: total indirect | 0.0099 | [0.0032, 0.0230] |
| Women: total effect | −0.0010 | [−0.0125, 0.0107] |
| Men: indirect via body self-esteem | 0.0083 | [0.0028, 0.0165] |
| Men: indirect via EDE-Q8 | 0.0068 | [0.0011, 0.0158] |
| Men: total indirect | 0.0151 | [0.0062, 0.0282] |
| Men: total effect | 0.0116 | [−0.0093, 0.0310] |
| Difference of the within-group contrasts (women minus men) | 0.0041 | [−0.0066, 0.0157] |

## Table S19. Log-transformed BMI

**Table S19a. Model fit with BMI and with log(BMI) as the predictor**

| **Model** | **Chi-square** | **df** | **CFI** | **TLI** | **RMSEA** | **SRMR** |
| --- | --- | --- | --- | --- | --- | --- |
| Analytic sample (BMI) | 288.21 | 102 | 0.924 | 0.902 | 0.083 | 0.048 |
| Including extreme (BMI) | 280.43 | 102 | 0.926 | 0.905 | 0.081 | 0.046 |
| Including extreme (log BMI) | 281.71 | 102 | 0.927 | 0.905 | 0.081 | 0.047 |

*Note. The first row corresponds to the analytic sample (BMI ≤ 50 kg/m²); the other two rows correspond to the sample that includes the extreme observations.*

**Table S19b. Standardized effects (β) and p-values with BMI and with log(BMI)**

| **Group** | **Effect** | **Analytic sample (BMI): p** | **Including extreme (BMI): p** | **Including extreme (log BMI): p** | **Analytic sample (BMI): β** | **Including extreme (BMI): β** | **Including extreme (log BMI): β** |
| --- | --- | --- | --- | --- | --- | --- | --- |
| Women | a1 (BMI → body self-esteem) | < .001 | .053 | .007 | −0.265 | −0.122 | −0.160 |
| Women | a2 (BMI → EDE-Q8) | < .001 | .010 | < .001 | 0.358 | 0.190 | 0.260 |
| Women | b1 (body self-esteem → depression) | < .001 | < .001 | < .001 | −0.300 | −0.293 | −0.293 |
| Women | b2 (EDE-Q8 → depression) | < .001 | < .001 | < .001 | 0.367 | 0.327 | 0.342 |
| Women | c′ (direct effect) | < .001 | .076 | .015 | −0.181 | −0.108 | −0.138 |
| Women | Indirect via body self-esteem | < .001 | .075 | .022 | 0.080 | 0.036 | 0.047 |
| Women | Indirect via EDE-Q8 | < .001 | .029 | .004 | 0.131 | 0.062 | 0.089 |
| Women | Total indirect effect | < .001 | .024 | .002 | 0.211 | 0.098 | 0.136 |
| Women | Total effect | .597 | .859 | .977 | 0.029 | −0.010 | −0.002 |
| Men | a1 (BMI → body self-esteem) | .001 | .001 | .001 | −0.207 | −0.207 | −0.197 |
| Men | a2 (BMI → EDE-Q8) | < .001 | < .001 | < .001 | 0.263 | 0.263 | 0.276 |
| Men | b1 (body self-esteem → depression) | < .001 | < .001 | < .001 | −0.304 | −0.304 | −0.305 |
| Men | b2 (EDE-Q8 → depression) | .010 | .010 | .008 | 0.196 | 0.196 | 0.202 |
| Men | c′ (direct effect) | .765 | .765 | .578 | −0.027 | −0.027 | −0.048 |
| Men | Indirect via body self-esteem | .014 | .014 | .013 | 0.063 | 0.063 | 0.060 |
| Men | Indirect via EDE-Q8 | .059 | .059 | .038 | 0.052 | 0.052 | 0.056 |
| Men | Total indirect effect | .005 | .005 | .002 | 0.115 | 0.115 | 0.116 |
| Men | Total effect | .249 | .249 | .372 | 0.087 | 0.087 | 0.068 |

*Note. Unstandardized coefficients of the log(BMI) model refer to a one-unit change in log(BMI) and are not comparable with those of the models with BMI; the comparison therefore relies on standardized effects.*

**Table S19c. Classification of the mediation pattern with log(BMI)**

| **Group** | **Total indirect (b)** | **p indirect** | **Direct c’ (b)** | **p direct** | **Total effect (b)** | **p total** | **Classification (Zhao et al., 2010)** |
| --- | --- | --- | --- | --- | --- | --- | --- |
| Women | 0.430 | .002 | −0.435 | .015 | −0.005 | .977 | Competitive mediation; total effect not significant |
| Men | 0.410 | .002 | −0.169 | .578 | 0.240 | .372 | Indirect-only mediation; total effect not significant |

**Table S19d. Wald tests of differences between women and men with log(BMI)**

| **Contrast** | **Estimate** | **SE** | **z** | **p** | **95% CI** |
| --- | --- | --- | --- | --- | --- |
| BMI → Body self-esteem (a1): women minus men | 4.180 | 5.009 | 0.83 | .404 | [−5.636, 13.997] |
| BMI → EDE-Q8 (a2): women minus men | 1.424 | 5.034 | 0.28 | .777 | [−8.443, 11.291] |
| Body self-esteem → Depression (b1): women minus men | −0.001 | 0.006 | −0.13 | .898 | [−0.012, 0.011] |
| EDE-Q8 → Depression (b2): women minus men | 0.004 | 0.007 | 0.63 | .531 | [−0.009, 0.018] |
| Direct effect (c’): women minus men | −0.266 | 0.353 | −0.75 | .451 | [−0.957, 0.425] |
| Indirect via body self-esteem: women minus men | −0.065 | 0.107 | −0.60 | .547 | [−0.275, 0.145] |
| Indirect via EDE-Q8: women minus men | 0.085 | 0.137 | 0.62 | .534 | [−0.183, 0.353] |
| Total indirect effect: women minus men | 0.020 | 0.191 | 0.11 | .915 | [−0.353, 0.394] |
| Total effect: women minus men | −0.246 | 0.323 | −0.76 | .447 | [−0.878, 0.387] |
| Within women: indirect via EDE-Q8 minus via body self-esteem | 0.133 | 0.097 | 1.37 | .169 | [−0.057, 0.323] |
| Within men: indirect via EDE-Q8 minus via body self-esteem | −0.016 | 0.121 | −0.14 | .892 | [−0.253, 0.220] |
| Difference of the within-group contrasts (women minus men) | 0.150 | 0.155 | 0.97 | .334 | [−0.154, 0.453] |

*Note. Estimates refer to unstandardized coefficients in log(BMI) units.*

**Table S20a. Sensitivity analysis for single paths (α = .05, two-sided, power = .80)**

| **Group** | **n** | **Minimum detectable correlation (80% power)** | **Minimum detectable f-squared for one coefficient in the depression equation (3 predictors)** |
| --- | --- | --- | --- |
| Women | 398 | 0.140 | 0.020 |
| Men | 264 | 0.171 | 0.030 |
| Total | 662 | 0.109 | 0.012 |

*Note. Minimum detectable correlation and minimum detectable f² for one coefficient in the depression equation with three predictors.*

**Table S20b. Approximate power to detect an indirect effect a × b for standardized paths a (rows) and b (columns)**

| **Group** | **n** | **Standardized a** | **b = 0.1** | **b = 0.15** | **b = 0.2** | **b = 0.25** | **b = 0.3** |
| --- | --- | --- | --- | --- | --- | --- | --- |
| Women | 398 | 0.10 | 0.26 | 0.44 | 0.50 | 0.51 | 0.51 |
| Women | 398 | 0.15 | 0.44 | 0.73 | 0.84 | 0.85 | 0.85 |
| Women | 398 | 0.20 | 0.50 | 0.84 | 0.96 | 0.98 | 0.98 |
| Women | 398 | 0.25 | 0.51 | 0.85 | 0.98 | 1.00 | 1.00 |
| Women | 398 | 0.30 | 0.51 | 0.85 | 0.98 | 1.00 | 1.00 |
| Men | 264 | 0.10 | 0.14 | 0.25 | 0.33 | 0.36 | 0.37 |
| Men | 264 | 0.15 | 0.25 | 0.47 | 0.62 | 0.68 | 0.69 |
| Men | 264 | 0.20 | 0.33 | 0.62 | 0.82 | 0.89 | 0.91 |
| Men | 264 | 0.25 | 0.36 | 0.67 | 0.89 | 0.97 | 0.98 |
| Men | 264 | 0.30 | 0.37 | 0.68 | 0.90 | 0.98 | 1.00 |

*Note. Power approximated as the product of the power of paths a and b (joint significance test; MacKinnon et al., 2002).*

# 5. Supplementary figure

**Figure S2. Unstandardized direct, indirect and total effects of BMI on depressive symptoms in women and men**

*Note. Points are unstandardized estimates and lines are 95% confidence intervals based on robust standard errors (delta method for indirect and total effects). BMI is expressed in kg/m².*

# 6. References

Belsley, D. A., Kuh, E., & Welsch, R. E. (1980). Regression diagnostics. Wiley.

Bonett, D. G., & Wright, T. A. (2000). Sample size requirements for estimating Pearson, Kendall and Spearman correlations. Psychometrika, 65(1), 23-28. https://doi.org/10.1007/BF02294183

Champely, S. (2020). pwr: Basic functions for power analysis (R package version 1.3-0). https://doi.org/10.32614/CRAN.package.pwr

Chen, F. F. (2007). Sensitivity of goodness of fit indexes to lack of measurement invariance. Structural Equation Modeling, 14, 464-504.

Hoenig, J. M., & Heisey, D. M. (2001). The abuse of power: The pervasive fallacy of power calculations for data analysis. The American Statistician, 55(1), 19-24. https://doi.org/10.1198/000313001300339897

Jorgensen, T. D., Pornprasertmanit, S., Schoemann, A. M., & Rosseel, Y. (2026). semTools: Useful tools for structural equation modeling (R package version 0.5-8). https://CRAN.R-project.org/package=semTools

MacKinnon, D. P., Lockwood, C. M., Hoffman, J. M., West, S. G., & Sheets, V. (2002). A comparison of methods to test mediation and other intervening variable effects. Psychological Methods, 7(1), 83-104. https://doi.org/10.1037/1082-989X.7.1.83

Preacher, K. J., & Hayes, A. F. (2008). Asymptotic and resampling strategies for assessing and comparing indirect effects in multiple mediator models. Behavior Research Methods, 40, 879-891.

R Core Team. (2025). R: A language and environment for statistical computing. R Foundation for Statistical Computing. https://www.R-project.org/

Rosseel, Y. (2012). lavaan: An R package for structural equation modeling. Journal of Statistical Software, 48(2), 1-36. https://doi.org/10.18637/jss.v048.i02

Zhao, X., Lynch, J. G., & Chen, Q. (2010). Reconsidering Baron and Kenny: Myths and truths about mediation analysis. Journal of Consumer Research, 37(2), 197-206. https://doi.org/10.1086/651257
